# Efficacy of Enhanced HIV Counseling for Risk Reduction during Pregnancy and in the Postpartum Period: A Randomized Controlled Trial

**DOI:** 10.1371/journal.pone.0097092

**Published:** 2014-05-13

**Authors:** Suzanne Maman, Dhayendre Moodley, Heathe Luz McNaughton-Reyes, Allison K. Groves, Ashraf Kagee, Prashini Moodley

**Affiliations:** 1 University of North Carolina at Chapel Hill, Chapel Hill, North Carolina, United States of America; 2 University of KwaZulu-Natal, Durban, South Africa; 3 Stellenbosch University, Matieland, South Africa; Burnet Institute, Australia

## Abstract

**Introduction:**

Pregnancy and the postpartum period present important intervention opportunities. Counseling can leverage the motivation women have during this time to change behaviors that may negatively affect their health and the heath of their infants.

**Methods:**

Pregnant women attending an antenatal clinic in South Africa were randomly allocated to treatment (n = 733) and control arms (n = 747). Treatment arm participants received enhanced HIV pre- and post-test counseling, legal support and access to support groups at baseline, which occurred at the first antenatal visit, and then six and ten weeks postpartum. Control arm participants received standard HIV testing and counseling (HTC) and two postpartum attention control sessions. Outcomes were incidence of sexually transmitted infection (STI) by 14 weeks postpartum and past 30-day inconsistent condom use at 14 weeks and 9 months postpartum.

**Results:**

There were no intervention effects on incident STIs for either HIV-negative (adjusted risk ratio (aRR) 1.01, 95% CI 0^.^71–1^.^44) or HIV-positive participants (aRR 0^.^86, 95% CI 0^.^61–1^.^23). The intervention was associated with a 28% decrease in risk of past 30-day inconsistent condom use at nine-months among HIV-negative women (aRR 0^.^72,95% CI 0^.^59–0^.^88), but did not affect inconsistent condom use among HIV-positive women (aRR1.08; 95% CI 0.67–1.75).

**Discussion:**

An enhanced counseling intervention during pregnancy and the postpartum period can lead to reductions in inconsistent condom use among HIV-negative women. Results underscore the importance of the counseling that accompanies HIV HTC. More work is needed to understand how to promote and sustain risk reduction among HIV-positive women.

**Trial Registration:**

ClinicalTrials.gov NCT01683461

## Introduction

There has been a global effort to expand access to HIV testing and counseling for pregnant women due to the increased availability of effective biomedical interventions that reduce transmission of HIV from mothers to infants [1]–[3]. HIV counseling and testing (HCT) protocols were designed for clients seeking voluntary HIV counseling and testing services, and as such they were not tailored to be used in other health care settings such as antenatal care to address the decisions that women have to make in the context of pregnancy. There has been little empirical evidence to suggest how to effectively adapt HCT protocols for women during pregnancy [4]. Given that many women are presented with the opportunity for HCT for the first time in antenatal care there is a need to tailor HCT programs for antenatal care to capitalize on this prevention opportunity. There is also evidence that existing HCT protocols are not adequately addressing the needs of pregnant women. In a report commissioned by the South African Department of Health assessing the national pilot prevention of mother-to-child transmission (PMTCT) program, the authors described a strong focus of programs on the use of drugs to prevent HIV, with little understanding of the social and behavioral aspects of such an intervention [5]. Pregnancy and the postpartum period represent important opportunities to intervene with women to address their health and the health of their families. There is evidence that women are more motivated to address health issues during pregnancy and the postpartum period and it may be easier to change behaviors during such important transitional periods [6]. According to the Transtheoretical Model of Health Behavior Change, in order for people to progress from contemplating behavior change to preparation for and action to change behavior, they must get to a point where there pros of the change outweigh the cons [7]. Self-evaluation and environmental evaluation of the intended behavior change or realization that the target behavior is important not only to themselves but may also positively affect others, are instrumental in pushing individuals beyond contemplation to preparation for action and taking action on their intended behavior change [7]. The arrival of a new baby may influence women’s environmental evaluation and self-evaluation of a behavior that they had previously considered but not acted upon. Counseling can leverage the motivation women have during pregnancy and in the postpartum period to change behaviors that may negatively affect their health and the health of their infants. Accessing pregnant women through the health clinic is both a feasible and potentially cost-effective approach to intervening with them, particularly in the South African context where 92% of women have access to antenatal care [8], and where women return to clinics to immunize their babies at reasonably high rates [9]. Pregnant women in countries that are burdened by high sexually transmitted infections (STI) and HIV incidence are at risk for STI acquisition and HIV seroconversion during pregnancy and the postpartum period [10], [11]. The high rates of seroconversion and STI acquisition during pregnancy and the postpartum period necessitates increased prevention during this vulnerable period. The aim of the current study was to evaluate the effectiveness of an enhanced HIV testing and counseling intervention for HIV-positive and HIV-negative pregnant women on consistent condom use and incident STIs during pregnancy and the postpartum period.

## Research Design and Methods

### Ethics Statement

The study was approved by the ethical review committees at the University of North Carolina at Chapel Hill and the University of KwaZulu-Natal, Nelson Mandela School of Medicine. The protocol for this trial and supporting CONSORT checklist are available as supporting information; see Checklist S1 and Protocol S1.

### Design, Participants, Randomization and Assessment Procedures

The enhanced counseling intervention was evaluated using a Randomized Control Trial with participants randomly assigned to two arms (described in detail below): (1) the intervention arm, wherein participants received an enhanced counseling intervention; (2) the control arm, in which participants received standard of care counseling.

Participants were recruited from a primary health care clinic in Umlazi Township, Durban, South Africa. Umlazi, South Africa’s second largest township, is located in the province of KwaZulu Natal (KZN) which has the highest antenatal prevalence of HIV infection and highest infant mortality in South Africa [12]. Pregnant women who presented to the clinic for their first antenatal visit were eligible for screening. Inclusion criteria were: (1) at least 18 years old, (2) had never tested for HIV or had tested negative for HIV at least 3 months prior to recruitment, (3) attending first antenatal visit when HIV testing was offered; (4) had a primary intimate partner for at least the past 6 months, (5) planned to live in Durban for at least the next year, (6) planned to bring their infant to the clinic for immunization visits, (7) able to communicate in English or Zulu, and; (8) did not need care for a high risk pregnancy since such patients needed to be referred to a tertiary public health facility. Recruitment for the trial began in May 2008 and ended in June 2010.

Immediately after screening, eligible participants who provided written informed consent completed baseline structured computer-assisted personal interviews (CAPI) administered by trained staff in Zulu or English according to participant preference. Following completion of the interview, women were randomized to receive either the enhanced counseling intervention (intervention) or standard counseling and testing (control) by asking them to select an opaque sealed envelope that contained a piece of paper with a computer-generated assignment from a box. Randomization was performed in permuted blocks, and a 1∶1 allocation ratio was used.

**Figure 1:**
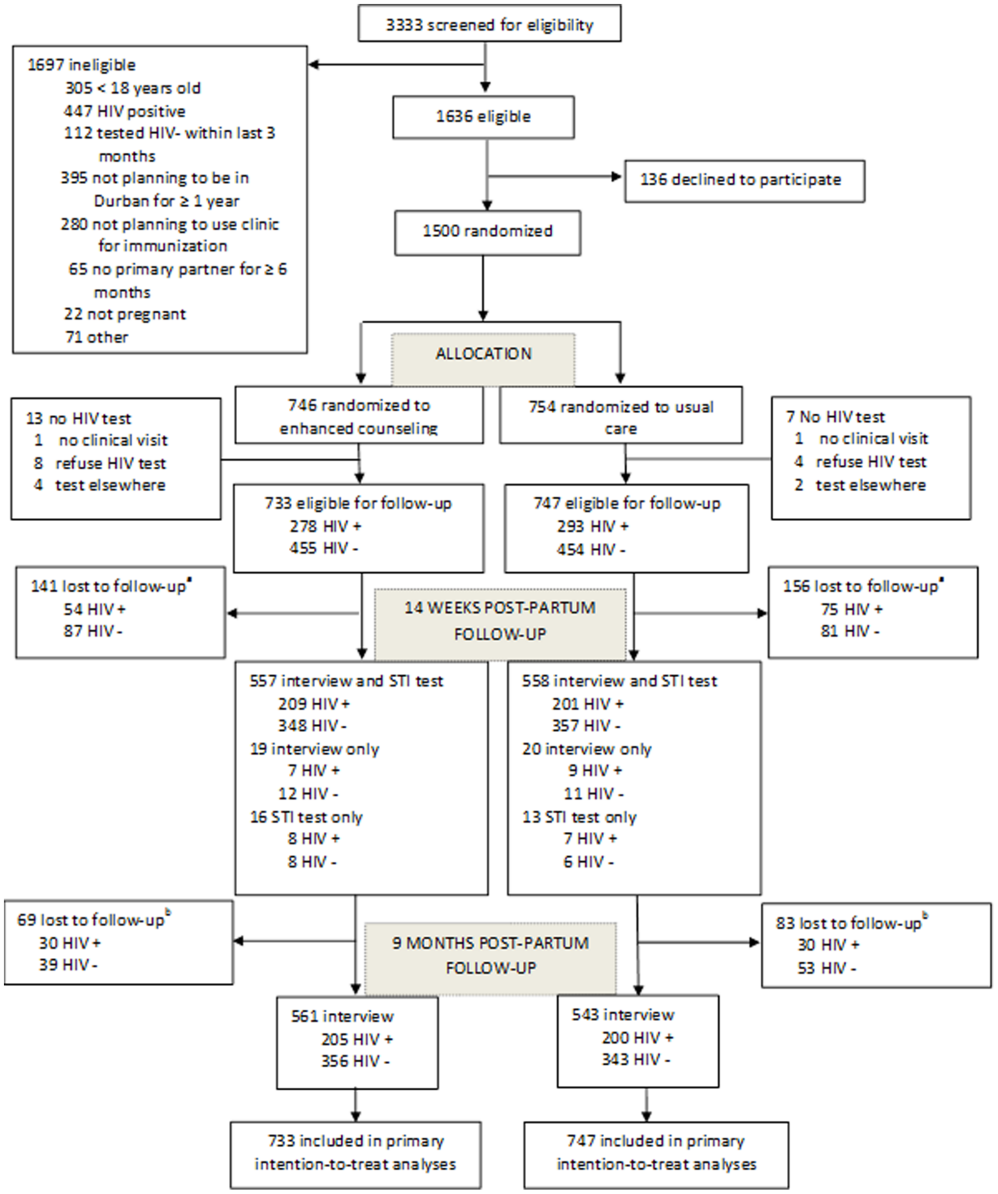
^a^Participants were considered lost to follow-up at 14 weeks post-partum if they did not complete an interview and were not tested for STIs. ^b^Participants were considered lost to follow-up at nine months if they did not participate in the 9 month post-partum interview and had either completed the interview or were tested for sexually transmitted infection at 14 weeks post-partum.

After randomization, study nurses, who were different from the interviewers, conducted clinical assessments of all participants that included: 1) antenatal care according to the South African Antenatal Care guidelines [13]; 2) collection of vulvo-vaginal swabs for the diagnosis of STIs, and; 3) HIV testing and pre- and post-test counseling. A participant provided a blood specimen from a finger prick for a rapid HIV test (Determine: Abbott Laboratories, Abbott Park, IL). If a reactive result was obtained with the Determine rapid HIV test, the participant’s HIV status was confirmed with a second rapid test (SmartCheck HIV 1&2, World Diagnostic Inc., Miami Lakes, FL, USA). In instances where a specimen was reactive to the Abbott kit, but negative on the SmartCheck, a blood specimen was sent for confirmation by a 4^th^ generation ELISA (HIV Combi, Roche Mannheim, Germany) performed at the Department of Virology, University of KwaZulu-Natal. A small number of women refused HIV testing (n = 12) or left the clinic prior to completing a clinical assessment (n = 2), precluding our ability to determine their HIV status. These women were deemed ineligible for follow-up because we were unable to provide them with any post-test counseling (either enhanced or standard of care) or determine which group to include them in (HIV-positive or HIV-negative) for analysis purposes. In addition, six women who left the clinic prior to the clinical assessment returned at a later date to be assessed, but reported having undergone HIV testing and counseling at another facility (n = 6); these women became ineligible to participate since they had received test-results and counseling from another source (see Figure 1. Study Flow).

Follow-up CAPI interviews were conducted with intervention and control arm participants at 14 weeks and 9 months postpartum. Follow-up STI testing was conducted at 14 weeks postpartum only. Women were reimbursed 70 South African Rand, equivalent to $8 USD at the 14 week and 9 month postpartum assessment visits.

### Interventions

Women randomized to the control arm received the standard of care in terms of HIV counseling and testing during pregnancy that was guided by the South African national guidelines [14] and by international guidelines [15], [16]. They received one pre-test counseling session and one post-test counseling session during pregnancy. In addition, to ensure that the number of clinic visits and nurse contact was similar across both arms (i.e., attentional control), women in the control arm met with a study nurse at 6 and 10-weeks postpartum; the focus of these sessions was on infant health. Unless women asked, nurses did not provide any additional risk reduction counseling to women.

Women randomized to the intervention (enhanced counseling) arm received the following services which differed from the standard counseling that women received in the control arm in several important ways: (1) a 15-minute video that women watched prior to antenatal clinical assessment that primed them for decisions that they would have to make around HIV testing, disclosure, and risk reduction. This video was developed by our team based on formative research we conducted in the clinic. The video was piloted with focus groups of pregnant and postpartum women from the clinic prior to use. (2) enhanced pre-test counseling that included risk assessment and discussion of the implications of HIV test results using more interactive techniques such a role plays; (3) enhanced post-test counseling that included discussion about sexual risk reduction, partner testing, family planning, and disclosure at their 6 and 10-week postpartum visits, and; (4) access to onsite legal support and support groups. The content of each post-test counseling session was tailored more specifically for HIV-positive and HIV-negative women. There was greater emphasis on disclosure and social support for the HIV-positive women. The counseling also included risk reduction for HIV-positive women. The emphasis for HIV-negative women was risk reduction to maintain their negative status. Women in the intervention arm met with the same counselor for all counseling sessions. In the control arm, women often met with different counselors for the pre- and post-test counseling sessions, depending on who was available.

Counseling was provided by the same nurse-midwives who provided the clinical care to women. Three nurse-midwives were trained as treatment arm counselors and two nurse-midwives were trained as control arm counselors. Intervention arm counselors received a two week counseling training course prior to the study and three booster training sessions during the study period that were facilitated by a staff psychologist. The same staff psychologist also facilitated a one week training for the two control nurses in the skills necessary for conducting the HIV pre- and post-test counseling sessions. To minimize contamination, all antenatal and postnatal services for women in the intervention arm were provided in a separate facility on the grounds of the clinic, while services for women in the control arm were provided within the regular antenatal facility.

### Measures and Outcomes

Participants reported their age, education, pregnancy history, relationship length, and cohabitation status. Following previous research in South Africa, we created a measure of socioeconomic status by using principal components analysis to derive a linear index from a series of asset ownership indicators and then categorized participants as belonging to the poorest 40%, middle 40%, or wealthiest 20% on the asset index scale [17]–[22]. Pregnancy characteristics included parity and gestational age at baseline. Sexually transmitted infection (STI) incidence and inconsistent condom use were the primary biological and behavioral outcomes for this study respectively.

### STI Incidence

Study nurses tested participants at baseline and at 14 weeks postpartum for three STI pathogens: *Trichomonas vaginalis*, *Neisseria gonorrhoea* and *Chlamydia trachomatis*. The BD ProbeTec ET Amplified DNA Assay (Becton Dickinson, MD, USA) employing Strand Displacement Amplification (SDA) was used for the direct detection of *Neisseria gonorrhoeae* and *Chlamydia trachomatis*. *Trichomonas vaginalis* was detected by means of an in house PCR. Results were made available to the study nurses between 2–3 weeks from testing. Participants who tested positive for any of these infections at baseline were appropriately treated. Women presenting with vaginal discharge syndrome or genital ulcer syndrome at time of assessment received syndromic management which comprised a combination of cefixime (400 mg), metronidazole (2 g), benzathine penicillin (2.4 mU intramuscular injection), erythromycin (500 mg every 6 hours for 7 days), and acyclovir (400 mg every 8 hours for 7 days). Women who were asymptomatic but tested positive for any of the STI pathogens were informed at their subsequent antenatal visit or telephonically contacted and asked to return to the clinic sooner if the next antenatal visit was scheduled for a later date. STI history at baseline and STI incidence at 14 weeks postpartum was coded as “1” for participants who tested positive for any of the three infection pathogens and as “0” for participants who tested negative for all of the three infection pathogens.

### Inconsistent Condom Use

At each assessment (baseline, 14 weeks and 9 months post-partum), participants were asked to list their last five sexual partners starting with their current partner. For each partner, participants were asked how many times they had had sex during the past 30 days with that partner and how many times they had used a condom during sex. A 30-day retrospective period was selected because research has shown reports of partner numbers and sexual events to be reliable over this interval [23]. Inconsistent condom use in the past 30 days was defined as failure to report use of condoms all of the time during sex in the past 30 days [24] and was coded as “1.” Consistent condom users as well as those who reported not having had any sex in the past 30 days were coded as “0.”

### Statistical Analysis

The study was powered to allow for all statistical analyses to be stratified by HIV-status because the content of the enhanced counseling intervention differed for these two groups. Based on a 9% difference in rate incident STIs among HIV-positive women in the intervention and control arms and an 8% difference among HIV-negative women, 279 HIV-positive and 295 HIV-negative participants per group were needed for 80% power. For consistent condom use, this sample size has the power to detect a difference between groups of 12% for HIV-positive women and 11% for HIV-negative women.

Data on 1,480 women (571 HIV-positive, 909 HIV-negative) were analyzed. All statistical analyses were performed using SAS software, version 9.3, using a pre-determined α level of P<.05. Baseline characteristics of HIV-negative and HIV-positive women in the enhanced counseling and usual care groups were compared using *X* ^2^ tests for categorical data and *t* tests for continuous data. Logistic regression was used to examine baseline predictors of drop-out at the 14 week and 9 month follow-up assessments as well as differential attrition across study conditions.

Intention-to-treat analyses were conducted for all outcomes. Multiple imputation was used to deal with missing data on covariates (≤2% for all covariates) as well as due to study drop-out [25], [26]. Imputation was performed separately for HIV-negative and HIV-positive women using a fully conditional specification (FCS) method [27], [28]. Sensitivity analyses were performed to determine whether the results of the primary analyses differed when using the non-imputed data, which included only those observations that completed the follow-up assessments, as compared to the imputed data. Both produced similar results with negligible differences in parameter estimates and standard errors; therefore, we report the results of the imputed analyses.

Unadjusted and adjusted analyses were performed, with adjustment for baseline scores on all outcomes, the length of the exposure time between baseline and the outcome assessment, demographic and pregnancy characteristics. We used a modified Poisson regression approach to estimate the effects of the intervention on incident STI at 14 weeks postpartum and inconsistent condom use at 14 weeks and 9 months postpartum (three separate outcome variables), with intervention effects expressed as relative risks with robust standard errors [29]. Models for each outcome were adjusted for: the baseline level of the outcome variable (i.e., baseline STI status/inconsistent condom use), the length of the exposure time between baseline and the outcome assessment, age, socioeconomic status, education, pregnancy history, gestational age, relationship length, and cohabitation status.

## Results

### Participant Flow

Participant flow is presented in Figure 1. Of 3,333 women screened, 1,636 (49.1%) met the eligibility criteria and 1,500 (91.7%) women were enrolled and randomized. Subsequent to randomization, 13 women in the treatment arm and 7 women in the control arm either did not complete a clinical visit, refused HIV testing or indicated that they had tested at another location between screening and baseline assessment, and were therefore not eligible for follow-up yielding a final baseline sample size of 1480 women (733 intervention and 747 control). The proportion of participants who tested positive for HIV at baseline in pregnancy was the same across study arms (39%; 278 treatment and 293 control arm participants, respectively).

### Baseline Characteristics

Table 1 characterizes the total, HIV-positive and HIV-negative samples by assigned condition. On average, at baseline women were 25^.^5 years of age, 26% lived with their partner, and the average duration of relationships was 4^.^5 years. There were no significant differences between treatment and control groups in demographic or pregnancy characteristics. At baseline, inconsistent condom use was the norm, with approximately 70% of HIV-positive and HIV-negative participants in both study arms reporting having had sex without a condom in the past 30 days. Within the HIV-positive sample, baseline STI prevalence rates were similar across the treatment and control arms (39%); however, within the HIV-negative sample, participants randomized to the treatment condition were more likely to have tested positive for an STI at baseline (31%) compared to participants randomized to the control condition (24%; p = 0^.^02).

**Table 1:** Baseline Participant Characteristics^a^.

	Total (n = 1480)			HIV+(n = 571)			HIV - (n = 909)		
	Intervention (n = 733)	Control (n = 747)	*p*	Intervention(n = 293)	Control (n = 278)	*p*	Intervention (n = 455)	Control (n = 454)	*p*
*Demographic characteristics*									
Maternal age (y)	25.3 (5.2)	25.7 (5.5)	0.24	26.3±4.9	26.6±5.3	0.52	24.8±5.3	25.1±5.5	0.36
Education level									
Primary school completed	310 (42.3)	313 (41.9)	0.79	131 (47.1)	149 (50.9)	0.63	179 (39.3)	164 (36.1)	0.52
Secondary school completed	376 (51.3)	383 (51.3)		122 (43.9)	120 (41.0)		254 (55.8)	263 (57.9)	
Socioeconomic status									
Middle	274 (37.4)	299 (40.0)	0.57	102 (36.7)	104 (35.1)	0.91	172 (37.8)	195 (43.0)	0.09
High	148 (20.2)	142 (19.0)		44 (15.8)	47 (16.0)		104 (22.9)	95 (20.9)	
Lives with partner	197 (26.9)	187 (25.0)	0.45	78 (28.1)	87 (29.7)	0.67	119 (26.2)	100 (22.0)	0.22
Length of relationship (y)	4.4 (4.1)	4.6 (4.1)	0.44	3.8±3.4	4.0±3.6	0.44	4.8±4.3	4.9±4.5	0.62
*Reproductive history*									
Gestational age (wks)	24.3 (6.3)	24.2 (5.8)	0.75	23.3±6.3	23.8±5.6	0.31	24.8±6.2	24.4±5.9	0.25
Previous pregnancies									
One	272 (37.1)	282 (37.8)	0.88	109 (39.2)	136 (46.4)	0.22	163 (35.8)	146 (32.2)	0.44
Two or more	206 (28.1)	201 (26.9)		83 (29.9)	78 (26.6)		123 (27.0)	123 (27.1)	
*Baseline sexual risk status*									
Positive STI test	247 (33.7)	223 (29.9)	0.17	107 (38.5)	114 (38.9)	0.92	140 (30.8)	109 (24.0)	0.02
Inconsistent condom use	498 (67.9)	522 (69.9)	0.42	192 (69.1)	206 (70.3)	0.75	306 (67.3)	316 (69.6)	0.45

a Data are expressed as No. (%), unless otherwise indicated. The p-value was calculated by t-test for continuous variables and chi square for categorical variables.

### Retention

At 14 weeks postpartum, 81% (n = 592) of treatment group participants and 79% (n = 591) of control group participants completed either a follow-up interview and/or were tested for incident STIs. At 9 months postpartum, 77% (n = 561) of treatment group and 72% (n = 538) of control group participants completed a follow-up interview. Overall, drop-out rates were higher among HIV-positive than among HIV-negative participants; 29% of HIV-positive participants had dropped out by 9 months as compared to 23% of HIV-negative participants (X^2^(1) = 6^.^59; p = 0^.^01). Study-retention was not related to treatment condition.

### Intervention Exposure

Among HIV-positive participants assigned to the enhanced counseling arm (n = 293), 60% attended both the six and ten week postpartum counseling sessions and 17% participated in only one postpartum session. Similarly, among HIV-negative participants assigned to the enhanced counseling arm (n = 455), 60% participated in both postpartum counseling sessions and 15% participated in one postpartum session. There were no harms or unintended effects to report in either arm.

### Primary Outcome Analyses

The number and percent of participants who reported inconsistent condom use are presented descriptively in Table 2. Results from the outcome analyses of treatment effects are presented in Table 3.

**Table 2:** Sexually transmitted infection and past 30-day inconsistent condom use with any partner across baseline, 14-week and 9-month post-partum follow-up^a^.

	14 weeks postpartum			9 months postpartum		
	n	%	p-value	n	%	p-value
Sexually transmitted infection^b^						
HIV-positive						
Treatment	214	22.9	0.32	–	–	–
Control	203	27.1		–	–	
HIV-negative						
Treatment	349	16.6	0.58	–	–	–
Control	358	15.1		–	–	
Inconsistent condom use^c^						
HIV-positive						
Treatment	209	11.5	0.06	197	15.2	0.65
Control	208	6.3		198	13.6	
HIV-negative						
Treatment	353	17.6	0.36	357	28.1	0.009
Control	360	20.3		337	37.4	

a Unimputed sample sizes and proportions for each group. P-values are based on chi-square tests of differences in proportions between intervention and control group. Sample size differs across groups, outcomes, and assessment periods due to attrition and missing data.

b Baseline STIs were treated so that incident STIs at 14 week follow-up could be evaluated. No STI testing was conducted at 9-month post-partum.

c Women who were not sexually active were coded as “0” for inconsistent condom use.

**Table 3:** Relative risk of incident sexually transmitted infection (STI) and past 30-day inconsistent condom use with any partner in the enhanced counseling (intervention) as compared to control group.

Outcome		
	RR (95% CI)	p-value
Incident STI at 14 weeks postpartum^a^		
HIV-positive	0.86 (0.61, 1.23)	0.42
HIV-negative	1.01 (0.71, 1.44)	0.95
Inconsistent condom use at 14 weeks postpartum		
HIV-positive	1.72 (0.96, 3.08)	0.07
HIV-negative	0.81 (0.59, 1.12)	0.20
Inconsistent condom use at 9 months postpartum		
HIV-positive	1.08 (0.67, 1.75)	0.73
HIV-negative	0.72 (0.59, 0.88)	0.001

Abbreviations: CI, confidence interval; RR, relative risk.

a Separate models were estimated for HIV-positive and HIV-negative participants using imputed data.

Note: *p<.05; **p<.01; ***p<.001. RR = relative risk. Separate log binomial regression models were estimated for HIV-positive and HIV-negative participants for each outcome. Relative risks denote the likelihood of the outcome in the enhanced counseling (intervention) group compared to the control group. Models were adjusted for: baseline levels of the outcome, assessment interval, age, socioeconomic status, education, pregnancy history, gestational age, relationship length, and cohabitation status.

### Incident Sexually Transmitted Infection

STI prevalence at baseline was significantly higher among HIV-positive women (39%) than among HIV-negative women (24%); X2 = 20^.^77(1), p<0^.^001). Similarly, STI incidence at 14 weeks postpartum was significantly higher among HIV-positive women (26%) than among HIV-negative women (17%, X2 = 18^.^55(1), p<0^.^001). There were no significant intervention effects on incident STI for either the HIV-positive or the HIV-negative subsamples. The proportion of HIV-positive women who tested positive for a sexually transmitted infection at 14 weeks follow-up was 23% in the treatment arm and 27% in the control arm (adjusted relative risk [5], 0^.^86; 95% CI,0^.^61–1^.^23). Among HIV-negative women, rates of incident STI infection were 17% in the treatment arm and 15% in the control arm (aRR, 1.01, 95% CI 0^.^71–1^.^44).

### Inconsistent Condom Use

At baseline, inconsistent condom use was high among both HIV-positive and HIV-negative women (69% overall prevalence). Across both groups, inconsistent condom use decreased substantially at 14 weeks postpartum (14% overall prevalence) and then increased again at 9 months postpartum (49% overall prevalence). We also note that, across treatment and control groups, overall decreases in the prevalence of inconsistent condom use were greater among HIV-positive women than among HIV-negative women (see Table 2). For HIV-positive women, treatment group assignment was not associated with significant differences in inconsistent condom use at 14 weeks or at 9 months. However, for HIV-negative women, treatment group was significantly negatively associated with inconsistent condom use (aRR 0^.^72,95% CI 0^.^59–0^.^88) at 9 months. There was a 28 percent decrease in the risk of past 30 day inconsistent condom use at 9 months among HIV-negative women in the intervention group as compared to HIV-negative women in the control group (p = 0.001).

## Discussion

The results of this randomized control trial indicate that there was an intervention effect for HIV-negative women with regard to inconsistent condom use at 9 months postpartum. HIV-negative women in the treatment arm reported less inconsistent condom use as compared to HIV-negative women in the control arm. Most HIV counseling and testing interventions have shown effect on risk behavior change among HIV-positive clients [30]. Decreasing risk behavior among HIV-negative clients of HIV counseling and testing programs has been a challenge. Most studies measuring the effect of HIV counseling and testing on behavior change have been conducted in non-pregnant populations. It is possible that the risk reduction counseling did leverage HIV-negative women’s motivation to keep themselves and their infants uninfected.

The change in risk behavior did not translate into differences in incident sexually transmitted infections among HIV-negative women, and we found no intervention effects on condom use or STIs for HIV-positive women. That may be related to a number of reasons. First, the effects we identified on inconsistent condom use among HIV-negative women in the treatment arm may have strengthened over time as women began to reengage in increased sexual activity later in the postpartum period. Thus, it is possible that we did not have a sufficiently long enough follow-up period to see the resulting decrease in STI incidence in this population. Second, the prevalence of sexually transmitted infections in South Africa is so high that even with the most powerful intervention, it is challenging to effect change without also mounting a campaign to treat the partners of the women [31]. Finally, it may also be that the services that women received in the control arm was sufficiently powerful to facilitate a change in the identification and treatment of STIs because all women had ongoing access to clinical care by nurses throughout the study period.

The intervention did not lead to a change in inconsistent condom use among HIV-positive women. Other behavioral interventions have also failed to show an effect on condom use among HIV-positive women [32]. It may be that HIV-positive women who are pregnant are primarily concerned with reducing the transmission risk to their infants and treating their own infection. Attention to risk reduction to reduce the risk to uninfected partners, or to minimize the risk of re-infection may not be motivators to behavior change for these women. HIV-positive women may also be less likely to use a condom when they have an HIV-positive partner [32]. The reason there was no change among the HIV-positive women could also be due to the fact that the counseling protocols for the HIV-positive and HIV-negative women were tailored and somewhat different. While the protocols for the HIV-positive women did include an emphasis on risk reduction, these counseling protocols also covered other issues that present challenges for HIV-positive women, including for example, infant feeding and disclosure.

The study is not without limitations. Women had to have a primary partner, defined as someone they had been with for at least 6 months, to be enrolled in the study and the average length of relationship was four years or longer across all study participants. The intervention may have worked differently in a population of women who were in less stable partnerships. Retention may have limited our ability to detect an intervention effect. We experienced loss to follow up in both arms, despite our best efforts at retention. We achieved 77% retention in the treatment group and 72% retention in the control groups at 9 months. In an urban, highly mobile setting like Durban, this level of retention in a large trial is comparable to what other behavioral trials have achieved [33]. There were 22% of HIV-positive women and 25% of HIV-negative women in the treatment arm who were not exposed to the postpartum counseling sessions, which may have led to an underestimate of the intervention effect. Women in the control arm had the option of seeking additional counseling beyond the pre- and post-test sessions that are part of the standard protocol. While we believe very few women accessed any additional counseling during or after pregnancy, this was not documented systematically. If women in the control arm received additional counseling beyond was is standard, it is possible that this may have contributed to behavior change among women in the control arm. Finally, the study was designed to evaluate the package of services in the intervention arm, and as such we are not able to determine what specific components of the intervention were responsible for the intervention effect.

Nonetheless, we demonstrated that it is possible to increase the consistency of condom use among HIV-negative women with this enhanced model of HIV counseling and testing for pregnant women. These results underscore the importance of the counseling that accompanies HIV testing for women during pregnancy. The results also point to a number of additional studies that are needed to determine how to motivate change in condom use among HIV-positive women. Additional research could help determine how HIV-positive women balance the challenges that they face post-diagnosis including disclosure, infant feeding, and risk reduction to develop counseling protocols that promote risk reduction while acknowledging these other challenges. Additional research could also help understand all women’s sexual activity in the postpartum period, and how to motivate greater risk reduction earlier in the postpartum period. Finally, we need more research to develop effective strategies to engage male partners of the HIV-positive as well as HIV-negative women to promote condom use and STI treatment within the couple.

## Supporting Information

Checklist S1 **CONSORT Checklist.**
(DOC)

Protocol S1 **Trial Protocol.**
(RTF)
