# Supplementary material for: Efficacy of Enhanced HIV Counseling for Risk Reduction during Pregnancy and in the Postpartum Period: A Randomized Controlled Trial
Source: PLoS One. 2014 May 13;9(5):e97092. doi: 10.1371/journal.pone.0097092 (PMC4019645; doi:10.1371/journal.pone.0097092)
Supplement: Protocol S1 — Trial Protocol. (RTF) [file pone.0097092.s002.rtf]

PROTOCOL

South Africa Antenatal Posttest Support Study (SAHAPS):  Efficacy of HIV Post-test Support for Antenatal Care Attendees (ANC) in South Africa
Version 3.1 Dated March 8 2008

			Principal Investigators: 	Dr.  Suzanne Maman 
						Department of Health Behavior 
University of North Carolina Gillings School of Global Public Health (UNC)
							&
						Prof Dhayendre Moodley
						Department of Obstetrics and Gynaecology
						University of KwaZulu Natal (UKZN)

			Co-Investigators: 		Prof Hoosen M Coovadia
Office of the Victor Daitz Chair in HIV/AIDS Research Officer of the Director of Biomedical Science – HIVAN
							Centre for HIV/AIDS Networking (HIVAN), UKZN

							Prof Prashini Moodley
							Department of Medical Microbiology, UKZN
							
							Dr. Hannah Sebitloane
							Department of Obstetrics and Gynaecology, UKZN

							Dr. Ashraf Kagee
							Department of Psychology, Stellenbosch University
							
							Dr. Michael Sweat
							Social and Behavioral Interventions Department
							Johns Hopkins Bloomberg School of Public Health

							Dr. Shrikant Bangdiwala
							Department of Biostatistics, UNC
							

							Dr. Heath Luz McNaughton Reyes
							Department of Health Behavior, UNC
							 


A.  Specific Aims
This is a randomized controlled trial to examine the efficacy of an integrated model of HIV post-test support for women attending antenatal care (ANC) in South Africa.  We anticipate that the intervention will significantly expand the preventive health benefits of PMTCT programs to the sexual partners and infants of women, while concurrently increasing the psychosocial benefits and minimizing the psychosocial harms associated with HIV testing and HIV serostatus disclosure for women. The study will be conducted at the Umlazi Section D clinic in Durban. There is currently a dramatic scale up of funding underway in Africa of HIV prevention services targeting antenatal women. For example, the U.S. President's Emergency Plan for AIDS Relief (PEPFAR) will provide $15 billion in prevention funding, and South Africa is one of the 15 designated countries where funds will be used to support a government initiative to provide all pregnant women access to ARVs1. However, in South Africa, where more than 85% of pregnant women deliver in a health facility2 and the average number of ANC visits per woman exceeds the national target2, there is still sub-optimal uptake of voluntary counseling and testing (VCT) and antiretrovirals (ARVs)3.   It is therefore crucial that feasible strategies be identified to better integrate prevention of mother to child transmission (PMTCT), HIV VCT, and reproductive health programs.  The proposed intervention is designed to tailor VCT for the ANC setting and provide a continuum of psychosocial support for pregnant women through:  (1) a standardized health education video before HIV pre-test counseling; (2) HIV pre- and post-test counseling sessions that prepare women for decisions related to testing, serostatus disclosure and ARV prophylaxis and help women plan strategies for sexual risk behavior change; (3) two additional post-test counseling sessions which coincide with 6 and 10-week postpartum visits and focus on legal education and referral, partner testing, sexual risk behavior change and family planning decisions and; (4) an active referral system to post-test support groups run by a clinically trained staff psychologist.  The feasibility and acceptability of the intervention has been established by our team through preliminary studies.  The primary specific aims are:

(1)	To describe the demographic, social and psychological factors that affect sexual risk behaviors, partner uptake of VCT, family planning intention, infant feeding choices, and HIV serostatus disclosure to sexual partners, among HIV-positive and HIV-negative women attending the Umlazi Section D ANC clinic in Durban, South Africa. 

(2)	To conduct a randomized controlled trial of HIV VCT plus enhanced posttest support as compared to standard HIV VCT among HIV-positive and HIV-negative pregnant women in the Umlazi Section D ANC clinic to determine the independent effect of the intervention on the outcomes listed below, and to determine whether HIV-serostatus to sexual partners influences these intervention effects:
2.1 	Sexual risk of HIV as measured by:  STI incidence (Trichomonas vaginalis, Neisseria gonorrhea and Chlamydia), consistent and correct condom use among HIV-positive and HIV-negative women; and partner uptake of HIV VCT among HIV-positive women.  
2.2. 	Factors that affect mother-to-child transmission of HIV including: acceptance of HIV VCT among HIV-positive and HIV-negative women; acceptance of ARVs, adherence to national infant feeding guidelines, and family planning use among HIV-positive women.
2.3. 	Psychosocial outcomes of PMTCT participation as measured by: perceived social support, emotional distress and partner violence among HIV-positive and HIV-negative women.  
2.4 	With the addition of the legal education and referral component to the intervention study, we will also measure women's awareness of legal rights, awareness of legal recourse, perceived legal needs, uptake of legal assistance and outcome of seeking legal assistance within the 12 month study period. 

(3)	 Additionally, our secondary aim is to compare the following operational outcomes across study arms:  (a) counseling time required; (b) the proportion of women returning for ongoing counseling at 6 and 10 weeks; (c) client's perceived quality of services; (d) the cost of service provision and opportunity costs incurred by clients.
   


Our collaboration includes a multidisciplinary team of researchers from the U.S. and South Africa who will join efforts to test a culturally appropriate and feasible intervention designed to overcome limitations and expand the preventive health benefits of PMTCT programs. Results from this intervention study will be generalizable to other regions of sub-Saharan Africa that are faced with similar challenges related to HIV prevention.


B.  Background and Significance:

B.1. As South Africa leads the movement in sub-Saharan Africa to establish PMTCT programs, strategies are now needed that can strengthen and expand the benefits of these programs: South Africa is experiencing one of the largest and fastest growing HIV epidemics in the world.4 AIDS is the leading cause of death in South Africa.  Almost 25% of all South African children under age 15 have lost at least one parent to AIDS.5  Sixty-percent of all HIV-infected adults acquire their infection before the age of 25. Based on data from ANC sentinel surveillance sites and population-based studies, women between the ages of 25-29 years are most severely affected with rates between 32-34%.6,7 Forty-six percent of women seeking ANC services at King Edward VIII Hospital in KwaZulu-Natal, the proposed site for this study, are infected with HIV.  High prevalence of sexually transmitted infections (STIs) in South Africa is almost certainly one of the factors driving the explosive spread of HIV infection. Prevalence data on STIs among pregnant women from KwaZulu-Natal indicate that 52% of women had at least one STI, 12.9% were diagnosed with Chlamydia, 7.8% were diagnosed with Gonorrhea, and 41.4% were diagnosed with Trichomoniasis8.  It is estimated that in the absence of a comprehensive PMTCT program as many as 70,000 infants in South Africa are infected with HIV each year.4 There has been a global call to increase access to antiretrovirals (ARVs) that are widely available in the developed world and that have proven effective at reducing mother to child transmission of HIV. Nowhere has this call for increasing access to ARVs been more loudly voiced than in South Africa.  The feasibility of a facility-based intervention such as the distribution of ARVs for PMTCT is high in South Africa, given that a very large proportion of women deliver their babies in health care facilities.  In KwaZulu-Natal, for example, it is estimated that 83.6% of births were hospital or clinic-based deliveries.2 The average number of ANC visits per client in KwaZulu-Natal is also high at 4.3, exceeding the national target of 3 visits per woman.2  In May, 2001 the South African Department of Health launched a national pilot PMTCT program in health care facilities in nine provinces.  The PMTCT programs offer voluntary counseling and rapid on-site testing for HIV, single dose of Nevirapine (NVP) to HIV positive mothers and their infants (HIVNET 012), and infant formula free of charge to mothers who choose not to breastfeed. By October, 2002 the national government announced steps to initiate the HIVNET 012 protocol for all HIV-positive women in state hospitals.   There is currently a national effort to expand women's access to ARVs after pregnancy and delivery, although to date the implementation of these programs have been limited in scope.   

B.2. Low uptake of PMTCT programs by women is a major barrier to their implementation.  Several PMTCT clinical trials have demonstrated a 37-50% reduction of MTCT using a short course of zidovudine9-11 and/or nevirapine12.  The establishment and scaling up of PMTCT programs in resource-constrained settings is a high priority for many funding agencies.  In 2002, President Bush launched the $500 million Mother-and-Child HIV Prevention Initiative designed to prevent mother-to-child transmission.  Rapidly scaling up PMTCT services is a key objective of the President's $15 billion PEPFAR initiative, with South Africa being one of the countries in sub-Saharan Africa designated to receive funds.1  However, there are a number of barriers to women's participation in PMTCT programs that may limit the widespread success of these programs.  In a wide range of African settings women's low uptake of PMTCT programs remains a persistent challenge.13-17  In order to benefit from interventions that can reduce mother to child HIV transmission women must be willing to be tested for HIV, and if they are HIV infected they must also be willing to accept and adhere to available ARV prophylaxis.  The experience of KwaZulu-Natal in rolling out PMTCT programs illustrates the way in which women's low uptake of PMTCT services compromises the program's ability to achieve their desired outcomes.  A total of 233,074 pregnant women received pre-test counseling for the period between June, 2001 through March, 2004, of whom 179,211 (77%) volunteered to test for HIV.  Of those who tested, 35% were HIV-positive.  Among both HIV-positive and HIV-negative women, approximately 90% received their HIV test results through post-test counseling.   Of the women who tested positive only 58% were issued with a single dose of NVP at the antenatal clinic.  In a report developed by the director of the KwaZulu-Natal (KZN) Provincial PMTCT program, Dr. Moodley (the co-PI on this study), the major challenge to implementing the PMTCT programs in all sites was the sub-optimal uptake of ARVs.  The KZN PMTCT report concludes that women need ongoing support to overcome barriers to enrolling in PMTCT programs.    

B.3. Involvement and support from male partners are critical to improving women's uptake of ARVs for PMTCT:  In Africa, it has been well documented that women often lack the power to make independent decisions with regard to their health care and the health care of their children18-20.  For example, we learned from research we conducted in Tanzania that women often feel the need to “seek permission” from a partner prior to HIV testing.21 Yet, it is also recognized that men in sub-Saharan Africa are often not involved in women's antenatal care.  ANC clinics are generally regarded as women's spaces, where men do not feel comfortable.22  This gap between women's inability to make independent health-related decisions and the lack of male involvement in ANC services represents a major challenge for PMTCT programs.  It is now widely acknowledged that partner support and involvement are critical for the implementation of PMTCT programs.23-25 The optimal uptake and adherence to PMTCT prevention programs is difficult for women whose partners are neither aware of nor supportive of their participation.  Involvement and support from partners has also been reported as the most important correlate of uptake and adherence to formula feeding for HIV-infected women in sub-Saharan Africa.26-29 Based on the experience of UNICEF PMTCT pilot studies in 11 countries (9 in sub-Saharan Africa), it was found that male involvement and support was critical to improving women's uptake of core PMTCT service, including the decision to test, returning for test results, correctly taking ARV drugs and choosing and carrying out an infant feeding method.  There is little empirical evidence on effective strategies to overcome the challenges of involving men in PMTCT programs. 

B.4. More research is needed to understand the role of disclosure in expanding the preventive health benefits of PMTCT programs:  In order to involve male partners in the PMTCT process, women must be willing to test and share HIV test results with their partners.  Between 16.7% to 64% of HIV-infected women in sub-Saharan Africa share HIV test results with their sexual partners.30-38  The lowest rates of HIV sersotatus disclosure to sexual partners are reported in studies among pregnant women.  Antelman reports that only 22% of women enrolled in an HIV perinatal transmission trial in Dar es Salaam, Tanzania disclosed HIV serostatus to their partner within 2-months, and only 40% disclosed after nearly 4-years of follow-up.30  Kilewo reports 16.7% of women enrolled in a perinatal trial in Tanzania shared test results with their partner during an 18-month study period.31  Higher rates of HIV serostatus disclosure are reported among women who received their results at a voluntary counseling and testing clinic. We found that 64% of HIV-positive women and 83% of HIV-negative women in a VCT clinic disclosed their serostatus to sexual partners 3-months after HIV testing in Tanzania36.  There is evidence from the literature that women face more barriers disclosing HIV serostatus to sexual partners than they do to other members of their social network.21,39,40  VCT protocols need to be tailored to address the challenges that pregnant women face disclosing their serostatus to their partners.

Through an expanded awareness of risk that comes with HIV serostatus disclosure, couples can take measures to protect themselves, which will ultimately reduce the prevalence of HIV infection among women of childbearing age.  Evidence from KwaZulu-Natal, the setting for the proposed research, indicates that women are the infected partner in approximately one-third of all serodiscordant couples.  Expanding awareness of risk to the seronegative partners of HIV infected women is an important extension of the preventive health benefits of PMTCT programs that has been overlooked. There is no guarantee, however, that disclosure of HIV serostatus will lead to subsequent sexual safety.  In the most recent and comprehensive review of studies from the U.S. examining the link between serostatus disclosure and sexual behavior change, Simoni was unable to demonstrate a consistent association between disclosure and safer sex, though as she points out this does not necessarily mean that disclosure is irrelevant to the practice of safer sex.41  She concludes that the key to safer sex, is whether the partners have explicitly discussed using protection and reached agreement about it.  Therefore, program strategies to support safe disclosure of results must be complemented by other strategies that encourage partner testing and communication about safe sex.  The lack of studies examining the link between serostatus disclosure and behavior change from settings outside the U.S. underscores the need for more research from developing country contexts with very high rates of HIV infection.  

Disclosure of HIV serostatus to sexual partners may enable couples to make informed reproductive health choices that could ultimately lower the number of unintended pregnancies among HIV-positive women.  One HIV-infected pregnant woman we interviewed in the pilot study we conducted of the proposed counseling said, “The counseling really assisted me.  When I tested and learned my results I felt like I was carrying a heavy luggage.  I didn't know how to start telling him.  So the counselor helped me to get the techniques of telling my husband. The counseling helped also because the problem I have (HIV-infection) does not allow me to keep delivering so it is a must that I share the test results with him so that we can decide on giving birth or not.” In some settings, disclosure is a prerequisite to enrollment in PMTCT+ programs, programs that provide ongoing access to ARVs to women, their infants and family members.  Experience of such a program from Kampala, Uganda found that failure to disclose was the major constraint to women's participation.42 Disclosure may also facilitate enrollment of women and their families in ongoing medical and psychosocial support programs.

B. 5.  Women experience a number of benefits and face some risks when they disclose their HIV status to their sexual partners.   A synthesis of the literature on the rates, barriers and outcomes of HIV serostatus disclosure, shows that for the majority of women in sub-Saharan Africa, disclosure of HIV test results to sexual partners was associated with less anxiety and increased social support.34,37,38,43,44  The reports of negative outcomes following HIV serostatus disclosure to partners, particularly physical assault, has caused some concern about partner notification policies.   In a review of the literature on HIV serostatus disclosure related violence in the U.S., Koenig found that the rates of physical violence following disclosure ranged between 0.5% to 4%.45  From sub-Saharan African studies, the rates of disclosure-related violence were higher on average and ranged between 3.5% and 14.6%31,34,46.  The definition and time frame for measuring violence varied in each study, so direct comparisons across studies are difficult.  Fear of negative social outcomes, including violence, was the most common barrier to disclosure reported by women in our study in Dar es Salaam, as well as in other studies from sub-Saharan Africa.30,33,35-37  Given the high baseline rates of violence in South Africa47 and the reports of disclosure-related violence by up to 14.6% of HIV-positive pregnant women, the need for supportive counseling among ANC clients to minimize these negative outcomes is critical.   Having conducted prior studies on the association between HIV and violence, we are now keen to test the efficacy of interventions that are designed to help women assess when disclosure to a partner is appropriate and safe, and interventions that support women through the disclosure process to minimize negative social outcomes including violence.

B.6. Integration of PMTCT, HIV VCT and family planning services averts HIV infections: While VCT and family planning counseling are standard components of PMTCT programs, the integration of these services has been a challenge, resulting in missed opportunities for HIV prevention counseling.  In order to reach the UNGASS goal of reducing the proportion of infants infected with HIV by 20% by 2005, and 50% by 2010, bold steps are required to better integrate and maximize the benefits of VCT and family planning counseling within PMTCT services.48,49  Women at PMTCT services are good candidates for family planning counseling in that they are all sexually active and fertile and the probability of future pregnancies among those women who do not use contraception is high (80%).50   In addition, most women in sub-Saharan Africa access health services only when they are pregnant, therefore reaching out to them at this point is critical.51   Contraceptive use among women in PMTCT programs is low (29%) and unmet need for family planning is high (25%).50  Based on theoretical models and empirical data, there is now evidence that effective contraception for HIV-infected women who do not wish to become pregnant prevents more infants from becoming infected than providing NVP for PMTCT, decreases the number of future orphans, saves lives of HIV-infected mothers who would have died in pregnancy or childbirth, and at a cost of $360 per death averted, is a cost-effective intervention.50,52,53  Despite the recognized benefit of integration, there are many missed opportunities for integrating family planning, VCT and PMTCT services.  Operational research data from sub-Saharan African show that only between a quarter to a half of HIV-positive and HIV-negative women enrolled in PMTCT receive family planning counseling.54  Training on VCT and family planning is given a low priority in PMTCT staff training protocols, and funding streams for these three interventions are usually separate, therefore, logistical integration of services also represents a major challenge.  The need for empirical data on the integration of these services provides the rationale for this trial.  

B.7. HIV VCT is an effective HIV prevention strategy that must be tailored for PMTCT programs:  There is evidence that HIV VCT is an effective HIV prevention approach for individuals who seek testing in free-standing VCT clinics, and particularly for HIV-infected individuals and couples.55 While the current standard protocols for HIV VCT are effective tools for behavior change, there are some shortcomings of these protocols that need to be addressed.  First, VCT protocols were designed for clients voluntarily seeking HIV counseling and testing services, and as such they need to be tailored for use in other health care settings, such as ANC clinics to specifically address the decisions that women have to make in the context of their antenatal care. While there is considerable current interest in adapting the standard VCT protocols for use in different service settings, there is little empirical evidence to suggest how this adaptation can be done effectively.56  Given that many women are presented with the opportunity for HIV VCT for the first time in ANC, there is a need to tailor VCT programs for ANC to capitalize on this prevention opportunity.  Furthermore, current HIV VCT protocols within PMTCT programs are not adequately addressing the psychosocial needs of women. In a report commissioned by the South African Department of Health assessing the national pilot PMTCT program, the authors describe a strong focus of programs on the use of drugs to prevent HIV, with little understanding of the social and behavioral aspects of such an intervention. 57  The report stressed the need to “provide on-going psychological and emotional support to HIV positive women as well as advice on disclosure.”  Evidence from a program in Johannesburg suggests that access to posttest support groups was highly valued by PMTCT clients and led to higher than average rates of HIV serostatus disclosure to sexual partners (69%), family planning (62%) and condom use (76%).  In this program women participated in support groups where disclosure, safer sex practices, and family planning were discussed.58  This evidence together with our experience piloting an enhanced model of HIV VCT for women underscores the need to test the efficacy of an integrated model of HIV post-test support for women enrolled in PMTCT programs. 

C: Preliminary Studies 
C.1. Experience of the study team: This study combines the efforts of investigators from The University of North Carolina School of Public Health in Chapel Hill, North Carolina, Johns Hopkins Bloomberg School of Public Health in Baltimore, Maryland and the Nelson R. Mandela School of Medicine in Durban, South Africa.  Preliminary research on HIV VCT and PMTCT from these collaborating institutions has set the stage for the proposed intervention trial.  The intervention study will be lead by Dr. Suzanne Maman, who has ten years of experience conducting HIV prevention research in sub-Saharan Africa.  Dr. Maman has served as PI on descriptive and intervention research studies related to HIV VCT and violence against women, including a pilot study of the proposed intervention among VCT clients in Dar es Salaam, Tanzania. Dr. Maman regularly serves as a technical advisor to international organizations such as WHO on gender-based aspects of HIV prevention and care.  Dr. Maman has expertise in qualitative research methods and is currently directing the qualitative assessment for NIMH Project ACCEPT, a multi-site, community-randomized trial of VCT.  Dr. Maman recently moved from the Johns Hopkins Bloomberg School of Public Health to join the faculty at the University of North Carolina at Chapel Hill in the Department of Health Behavior and Health Education.   As this is Dr. Maman's first R01 submission, she will be supported by a team of experienced Co-PI's and Investigators from both institutions.  Dr. Moodley will serve as the co-PI from the South African team.  Dr. Moodley is the Director of the Women's Health and HIV Research Unit at the Nelson Mandela School of Medicine, Dept. of Obstetrics and Gynecology.  She was seconded from the university to run the KwaZulu-Natal Provincial PMTCT program from 2000 to 2005.  She has served as the Principal Investigator on several large PMTCT clinical trials at King Edwards Hospital, including the South African Intrapartum Nevirapine Trial (SAINT) and the UNAIDS sponsored multicenter PETRA trial.   She currently has funding from Bristol-Meyers Squibb, Secure the Future, to enroll and follow a cohort HIV-positive and HIV-negative women from their first ANC visit until 12-months post-delivery.  She will be examining the seroconversion rate during pregnancy and breastfeeding, and the impact of the seroconversion on MTCT.  Dr. Moodley has been a strong advocate of programs that provide psychosocial support to HIV-positive and HIV-negative women in ANC settings.  
Dr. Michael Sweat from The Johns Hopkins University will serve as another Co-PI on the study.  Dr. Sweat has extensive background in the development and evaluation of behavioral interventions for HIV prevention in a wide range of developing countries.  Dr. Sweat conducted a cost effectiveness of PMTCT programs under different scenarios, to develop an equity analysis of PMTCT for different program delivery approaches, and to model the impact of ARVs on PMTCT.  This core research team will be supported by a number of other investigators from South Africa and the U.S. who have recognized expertise in perinatal HIV research (Dr. Sebitloane, South Africa), STIs (Prof Preshini Moodley  (South Africa), counseling (Dr. Kagee, South Africa) and analytical techniques for intervention research (Dr. Bangdiwala, USA).   We have also identified a Project Coordinator in South Africa who will be responsible for coordinating the intervention and research components of the trial (Elaine Smith). She has a Masters degree in Development Studies. The US research coordinator is Allison Groves who holds a Masters degree in Health Sciences from Johns Hopkins Bloomberg School of Public Health

C.2.   Pilot test of the enhanced HIV counseling approach: We completed a feasibility study of a model of HIV counseling similar to what we are proposing in this application, among 50 HIV-positive and 75 HIV-negative women at an HIV VCT clinic in Dar es Salaam, Tanzania (Suzanne Maman, PI).  The goal of this pilot study was to explore the feasibility and acceptability of an enhanced model of HIV counseling designed to help women overcome barriers and minimize negative outcomes of HIV serostatus disclosure to sexual partners. The study adapted current VCT protocols to include a greater focus on HIV serostatus disclosure and partner testing.  The enhanced model that we pilot tested included enhanced HIV pre-test counseling, and two enhanced post-test counseling sessions similar to the algorithm we are proposing in this trial.  We conducted in-depth interviews with all women enrolled in the pilot study 3-months after they tested and with the counselors who implemented the enhanced counseling protocols.  The following key findings emerged from the pilot study:  (1) Women felt that the development of detailed disclosure plans with the counselor was useful in helping them to overcome barriers to disclosure and developing communication techniques.  As one 38-year old, married, HIV-infected woman describes,” What assisted me was the counseling that I received. They gave me ideas of how to tell him…After I told him he was sympathetic.  Later on he said he would go for test.  He is trying to respect and care more for me.” (2) Several of the women enrolled in the pilot study were pregnant at the time of testing.  These women described specifically how the counseling helped them with issues related to their pregnancy, and making future reproductive health choices.  (3) Among women who had disclosed their HIV serostatus to their partner, over half reported that their partner had already tested or was planning to test.   From the perspective of the counselors we found that: (1) Counselors had no difficulty adopting the new counseling protocols for pre- and post-test sessions.  Counselors enjoyed using the role-plays with the clients and also appreciated the opportunity to have a second post-test counseling session with each client in order to follow-up on issues that were raised during the first post-test session; (2) The enhanced pre-test counseling took an average of 10-minutes longer to administer than the standard pre-test counseling session, and the two post-test sessions took an average of 30-minutes each to complete.  

C3.  VCT Studies:  The Multi-site Randomized Controlled Trial of the Efficacy of HIV VCT was the first randomized controlled clinical trial to test the efficacy of HIV VCT on behavior change (Michael Sweat, Co-PI). This study was the precursor to all of the studies that our team has since conducted on HIV VCT.  The design of the trial was a two-arm randomized controlled trial with baseline, six-and twelve-month assessments in Kenya, Tanzania and Trinidad.  Results of the trial indicate that VCT reduced unprotected intercourse with non-primary partners among both men and women getting tested and counseled.  Behavior change was greatest for HIV-infected participants.  The occurrence of most negative life-events as a consequence of HIV VCT was rare (0-4%); positive life events were more common (17-39%).  The PI for the proposed study (Suzanne Maman) has conducted follow-up studies at the Tanzania site based on the results of this behavioral intervention trial. Many publications were generated from the study which have informed this proposed project38,55,59 The team is also involved in an NIMH-funded community-randomized trial that is designed to test the hypothesis that communities receiving 2-1/2 years of community-based VCT relative to communities receiving 2-1/2 years of standard clinic-based VCT, will have significantly lower prevalence of recent HIV-1 infection.  The study is being conducted in five sites, Tanzania (PI: M. Sweat); South Africa (Vulindlela and Johannesburg), Zimbabwe and Thailand.  Dr. Maman is directing a qualitative assessment within the trial that consists of in-depth interviews with a cohort of community members in each site to describe how stigma changes over time in the communities, and will allow for an assessment of attitudes toward HIV-infected persons.  

C4.  HIV and Violence Studies: The PI of this proposal conducted a series of studies in collaboration with investigators from Muhimbili University College of Health Sciences in Tanzania on the associations between HIV and violence among women.  The first study was conducted to determine the role of violence as a risk factor for HIV and an outcome of testing and disclosure among women at a VCT clinic in Dar es Salaam.  Through this study we aimed to identify ways to mitigate violence associated with HIV testing, and disclosure of HIV serostatus. Three hundred and forty women were enrolled immediately after pre-test counseling. Women were followed prospectively and interviewed 3-months after testing.  Several key findings emerged: (1) The disclosure rate to primary partners 3-months after testing was 64% among HIV- positive women and 83% among HIV-negative women36 ; (2) The major reason for non-disclosure of HIV test results to partners, among women is fear of partner's reaction21; (3) Less than 5% of women reported any negative events following disclosure including violence and abandonment;  (4) 81.9% of HIV-negative women and 48.9% of HIV-positive women reported that their partner was emotionally supportive when they disclosed (p=.000)36; (5) When controlling for other demographic variables, the odds of reporting at least one physical or one sexually violent event was significantly higher among HIV-positive women as compared to HIV-negative women (physical violence OR: 2.63; 95% CI 1.23-5.63 and sexual violence OR 2.39; 95% CI 1.21-4.73); (6) The odds of reporting current partner violence was 10 times higher among young (<30 years) HIV positive women as compared to young HIV-negative women (OR 9.99; 95% CI: 2.67-37.37)60. Given the substantially higher rates of violence among young, HIV-infected women in this study, we are currently conducting an intervention trial for young men in Dar es Salaam, Tanzania (Suzanne Maman, PI).  The intervention addresses both HIV risk and violence through a combination of community-drama and peer support.   We will evaluate the intervention among a cohort of 800 young men through a matched untreated control group design with pre and post-intervention measures.  

C.5. Interventions to reduce perinatal HIV transmission: The South African team for the proposed study has extensive experience conducting biomedical, epidemiological and behavioral studies related to perinatal HIV transmission.  Dr. Moodley, the Co-PI on this study has been the PI on two important PMCT clinical trials at King Edward VII Hospital (KEH).   KEH was one of the sites of the Phase III Petra study. This was an UNAIDS-sponsored multi-center, randomized, double blind, placebo-controlled trial to evaluate efficacy, tolerance and effectiveness of three drug regimens using zidovudine in combination with lamivudine for the prevention of mother-to-child transmission of HIV.  The Durban site contributed 400 patients and had a follow-up greater than 90%61.  KEH was also a site for a large trial to compare the efficacy of two short course ARV regimens, known as the SAINT Trial funded by Boehringer Ingelheim 62.  1306 HIV-positive pregnant women were assigned to one of two arms: Arm A received 200mg of NVP during labor and 1 dose to mother and infant 24-48 hours post-delivery or and Arm B received multiple doses ZDV+3TC during labor and for 1 week to other and infant post delivery.  Results showed that both regimens were effective with results comparable to those observed with the HIVNET 012 regimen and the 3TC/ZDV regimen in the PETRA study.   The results of these trials demonstrate that efficacy of short course regimens for PMTCT make the possibility of policies for reducing mother-to-child transmission of HIV an attainable objective in many developing countries.  The research infrastructure established to support these large perinatal HIV transmission trials have remained in place at the Umlazi Section D ANC clinic and the Family Clinic, and will be utilized in the proposed intervention trial.  The experience of the South Africa team in conducting large, rigorous, longitudinal studies such as this underscores the research capacity for the proposed research.

C.6. Infant feeding studies:  HIV transmission during breastfeeding remains a challenge and this is currently an important focus of the Durban perinatal HIV group.  A cohort analysis of a recent vitamin A supplementation trial showed that feeding practices were associated with HIV transmission in the cohort of 549 mother-infant pairs. At three months, the prevalence of HIV infection was 18.8% in the 156 infants who never breastfed and 21.3% in 393 breastfed infants (p=0.5) 65. The prevalence was 24.1% in the 288 mixed-fed infants but only 14.6% in the 103 infants who were exclusively breastfed (p=0.03).  Dr. Moodley has been an investigator on these trials, and has been involved in ensuring that the results of these trials translate into the national guidelines for PMTCT in the Province of KwaZulu-Natal.   The national guidelines on infant feeding in South Africa are in accordance with international standards established by WHO and UNICEF.66  At the Umlazi Section D ANC all HIV-infected women currently receive general counseling on breastfeeding and safe alternatives from designated infant feeding counselors.  Counseling about feeding options include the health benefits and risks of breastfeeding and formula feeding.  This includes a discussion about the risk of HIV transmission through breastfeeding, the benefits of breastfeeding, the risks of mixed feeding, a description of replacement feeding options that are available, and an assessment of the feasibility and acceptability of exclusive formula feeding for women.67  The counselors assess the socioeconomic, environmental and home circumstances of HIV positive pregnant women during antenatal counseling to determine whether formula feeding will be safe and sustainable for each individual.  In this client-centered counseling model, women choose an infant feeding plan and they are supported in their choice by clinic staff.  The government of South Africa offers six months of free formula to all HIV-positive mothers who choose to use replacement feeding.  If women choose to breastfeed, they are advised to exclusively breastfeed on demand for the first four to six months of life and then abruptly wean the child and change to replacement feeding.  They are provided with a six-month supply of free infant formula at that point.   The infant feeding studies conducted by our team have lead us to recognize the growing need for improved counseling to address the infant feeding decisions that HIV-positive women must make in the context of their antenatal care. Data generated from studies such as these provide important knowledge that can inform the recommendations adopted as part of this trial.  With such extensive experience on infant feeding and risks associated with infant feeding, we can ensure that the HIV counselors trained as part of this trial are counseling women with the most up to date international best practices.  


D.  Research Design and Methods 

D1.  Overview: The first primary aim is to describe demographic, social and psychological factors that affect HIV sexual risk behaviors, partner uptake of HIV VCT, family planning intentions, infant feeding choices and HIV-serostatus disclosure to sexual partners among pregnant women attending the Umlazi Section D ANC clinic in Durban, South Africa. To meet this aim we will conduct ethnographic research with women at the Umlazi Section D ANC clinic and their male partners.  These qualitative data will provide us with insight into the determinants of these behaviors from the perspective of women and their male partners.  We will conduct 64 in-depth interviews with women from the ANC clinic.  We will also conduct 16 in-depth interviews with men who have been referred from their female partners at the ANC clinic.  
The second primary aim of the study is to conduct a randomized controlled trial of HIV enhanced post-test support as compared to standard HIV counseling among HIV-positive and HIV-negative women in the Umlazi Section D ANC clinic.  To do this we will randomize 1,495 women at Umlazi Section D ANC in Durban, South Africa to either a comparison or an intervention arm. Women randomized to the comparison arm will receive the standard WHO/CDC HIV pre-and post-test counseling and will have brief follow-up sessions with counselors 6- and 10-weeks postpartum. Women randomized to the intervention arm will receive: 1) a health education video immediately prior to HIV pre-test counseling; 2) pre and post-test counseling sessions that build on the standard protocols by providing more risk reduction counseling, more counseling on disclosure, and PMTCT-related decisions; 3) two additional post-test counseling sessions that coincide with the 6- and 10-week postpartum visits and focus on legal education and referral, partner testing, sexual behavior change and family planning; 4) an active referral to post-test support groups and 6) access to onsite legal support. Women in both arms will be administered a baseline survey at enrolment. Post-intervention assessment will occur at 14 weeks and 9-months post-partum. We will examine the independent effect of the intervention on primary outcomes related to sexual risk transmission of HIV, factors that affect mother-to-child transmission, and psychosocial outcomes of PMTCT participation for women.  We will also examine what influence HIV serostatus disclosure to sexual partners has on these effects.  See Figure 1.    
We have a secondary aim to compare operational outcomes across study arms.   We will compare  counseling time required, proportion of women returning for ongoing counseling at 6 and 10 weeks, clients perceived quality of services and the cost of service provision and opportunity cost incurred by client across the two arms.  This aim will be achieved through analysis of clinic records including counseling log forms and financial records. 

D.2. Study Site and Population

D.2.1. Umlazi Section D Clinic:  The Umlazi Section D Clinic is a primary health care (PHC) center situated 17km southwest of Durban. The clinic is staffed by 1 physician and 15 nurses, and offers services Monday through Friday. The VCT site within the ANC clinic has 4 full time trained counselors. The population of the surrounding Umlazi community is estimated at 400, 000 although some estimates indicate a higher population figure – up to 1 million people.  There are informal shack settlements in and around the clinic. It's a community that amplifies South Africa's already-significant HIV rate – Children orphaned by AIDS head almost 10% of the township's households. It is also estimated that more than 40% percent of the pregnant women are HIV positive. This community has one hospital, Prince Mshiyeni Memorial Hospital, which serves the surrounding area.  Most women are seen at the Umlazi Section D Clinic for all antenatal clinic visits, and then they deliver their babies at the Prince Mshiyeni Memorial Hospital.  All post-natal visits and well-baby visits are conducted at the Section D clinic.  The PHC clinic has an active 


PMTCT program.  Most women starttheir ANC visits at 28-weeks gestation, and women are usually offered testing at their first visit. Women are given standard HIV post test counseling according to WHO/CDC guidelines in the form of group counseling with 8-10 other women from the ANC clinic. Those who agree to be tested are tested for HIV according to the standard HIV testing algorithm used in the antenatal clinic. A participant provided a blood specimen from a finger prick for a rapid HIV test (Determine: Abbott Laboratories, Abbott Park, IL). If a reactive result was obtained with the Determine rapid HIV test, the participant's HIV status was confirmed with a second rapid test (Smart Check (WDI). In instances where a specimen was reactive to the Abbott kit, but negative on the SmartCheck, a blood specimen was sent for confirmation by an ELISA performed at the Department of Virology, University of KwaZulu-Natal.  All women are given standard HIV post-test counseling.  HIV infected women are: counseled on safe infant feeding options, allowed to make an independent choice on their preferred infant feeding method, given further information and support regarding their infant feeding choice, and have access to free formula if they opt to use replacement feeding. HIV-positive women are also offered a single dose of NVP according to the HIVNET 012 protocol. The drug is dispensed to women at 32-weeks of gestation.  All HIV-infected women receive an intrapartum dose of NVP (200mg) for self-administration at the onset of labor. When these women arrive at the hospital they are asked by the midwife in the labor ward whether she has taken her NVP dose. If women have not taken the dose then it is administered at that time.  All infants born to HIV-infected women receive a single dose (6mg if weighing 2kg or more and 2mg/kg for those weighing less than 2kg) of NVP within 24-hours of birth.  It is possible that changes to the nationally approved recommended regimen for ARV prophylaxis will occur during the course of the study, reflecting improvements in the standard of care in South Africa and concerns over resistance to Nevirapine among pregnant women. Should this happen we shall carefully document and track such changes. It should be noted that since this will occur consistently in both study arms randomization will help to control for any resulting biases from such a change in ARV regimen.   Women are invited to bring their partners in for testing.  After delivery, all post-partum women bring their babies for follow up according to the Expanded Program on Immunization (EPI) schedule at 6,10, and 14 weeks and 6,9 and 15 months to the PHC Clinic.     

D.2.2. Study Population:  There are about 9,000 first visit antenatal attendees per year in the ANC clinic at Umlazi Section D Clinic. Women range in age between 16 and 40 years. Approximately 25% are primagravid and the average gestational age at first visit is 28 weeks. Sixty percent of the women at the Umlazi ANC clinic have attended school for at least 8-10 years. Based on the most recent service statistics from the Umlazi Section D clinic, 21,369 women have been offered HIV testing since the program started in June, 2001. Of these women, 18,163 (85%) agreed to be tested, of whom, 8,173 (45%) were HIV infected. The transmission rate from mother to child is 34%, with approximately 95% transmission taking place during the pre and intrapartum period and the remainder post partum, primarily through breastfeeding. The prevalence in the clinic rose from 4% in 1990 to 46% in 2004.  

D.3. Ethnographic Research: To answer our first specific aim we will conduct qualitative interviews with women from the ANC clinic and the male partners of ANC clients.  The purpose of this ethnographic data is to describe factors that affect key study behaviors such as sexual risk behavior, ARV uptake, infant feeding choices, family planning intentions, partner testing and disclosure of serostatus to sexual partners from the perspective of women and their male partners.  This data will help inform the intervention and refine measures used in the quantitative instrument.  All in-depth interviews will be conducted in a private room in the ANC clinic, audiotaped, transcribed, translated and computerized for data analysis.  We will conduct qualitative research to: (1) Describe the factors that influence women's decisions about ARV acceptance, infant feeding, family planning, condom negotiation and to specifically assess what role HIV serostatus disclosure to partners plays with regard to each of these decisions.  We will conduct 64 in-depth interviews with women from the ANC clinic who have returned for the 6-week post-partum visit.  Among the 64 women we will purposively select both HIV-positive women and HIV-negative women to discuss the above topics.  To be eligible for these interviews women must be a client at the ANC clinic, must have been tested for HIV in an earlier ANC visit and must be able to identify a primary partner, which we will define as someone they have been with for at least 6-months.  (2) Describe male attitudes toward and determinants of participation in MTCT and uptake of HIV VCT: We will conduct 16 in-depth interviews with male partners of ANC clients. To ensure the safety of women, we will recruit these male partners through self-referrals from the women.  Ethnographers will ask women who report that they have shared their HIV test results with their partner and have not experienced negative outcomes as a result of this disclosure whether they would be willing to refer their partner to the clinic for an interview. (3) Pilot test the video:  We will pilot test the video with HIV-positive and HIV-negative women from the Umlazi Section D ANC clinic. We will conduct 5 focus group discussions (FGD) with 6-8 women each. Women who have been counseled and tested for HIV in the Umlazi Section D ANC clinic within the last one month will be recruited for each FGD.  The groups will watch the entire video in a private room within the ANC clinic.  After viewing the video the FGD facilitator will facilitate a discussion about the language and the content of the video. (4) Pilot test the counseling sessions: Through our pilot study among women in a VCT clinic we developed counseling tools, including the use of role-play scenarios and a disclosure-related violence structured counseling tool (described in section D.5.2.4).  For the purpose of the proposed study we will tailor these tools to antenatal women. We will use these tools in our counseling sessions, and will pilot our counseling program with 5 women in its entirety. Soon after the pilot sessions, women will be asked about acceptability, clarity and relevance of the counseling session; (5) Pilot test the interviewer administered questionnaire: Interviewer administered questionnaires (IAQ) has been the standard practice for other clinical trials conducted by the collaborating group at University of KwaZulu-Natal.  We will pilot our interviewer administered baseline survey 2 different ways. First, we will conduct cognitive interviews with 5 women to evaluate our baseline survey. During these interviews, we will ask each woman the questions within each section of the baseline survey. At the end of each section, we will ask each woman a series of questions about the content of the section in order to assess their understanding of each question, to determine whether the response categories are sufficient, and to evaluate whether questions need to be added, deleted or altered. Following the cognitive interviews, we will make any necessary changes to the baseline survey instrument. We will then pilot the baseline survey in its entirety with 15 women from the Umlazi Section D Clinic.

D.4. Participant processing and data collection procedures 

D.4.1. Subject recruitment and enrollment: To address our second specific aim, women will be recruited at the Umlazi Section D clinic.   Pregnant women who presented to the clinic for antenatal care are eligible for screening. Inclusion criteria were: (1) at least 18 years old, (2) had never tested for HIV or had tested negative for HIV at least 3 months prior to recruitment, (3) had a primary intimate partner for at least the past 6 months, (4) planned to live in Durban for at least the next year, (5) planned to bring their infant to the clinic for immunization visits, (6) able to communicate in English or Zulu, and (7) did not need care for a high risk pregnancy since such patients needed to be referred to a tertiary public health facility. Women who present to the clinic in labor will not be eligible to enroll. Women who have tested previously and are HIV positive are not eligible to enrol in the study. The person responsible for enrollment will meet with all ANC clients prior to their HIV pre-test counseling session. The survey interviewers will describe the purpose, procedures, risks and benefits of the study.  If women agree to participate, informed consent will be obtained prior to conducting the baseline survey. 

D.4.2. Baseline Survey:  Women who consent to participant in the trial will then complete a baseline survey facilitated by a survey interviewer using CAPI (computer assisted personal interview). The survey interviewer will asked the questions and enter the participant's response directly onto a laptop computer. This mode of data capture improves data quality as the data will be entered on the computer one time only.  Each computer will be password protected and locked up at the medical school at the end of each working day.  This interview will be conducted immediately after enrollment and prior to randomization and HIV pre-test counseling and will last approximately 45-minutes.  
D.4.3. STI Screening: We are proposing to use biological and behavioral measures of HIV risk in our study.  Behavioral and biological measures are relatively independent outcome measures that can provide different insight into the effectiveness of a behavior change intervention.  Neither measure can substitute or validate the other, and included together they provide a more complete measure of effectiveness.72 We propose using biologic markers of 3 STIs (Trichomonas vaginalis, Neisseria gonorrhea and Chlamydia) to enhance our understanding of the intervention effectiveness. These STIs are useful outcome measures for measuring intervention effectiveness because their prevalence and incidence is sufficiently high to warrant their use and because they are cured easily with a single treatment dose74.   Professor P Moodley, a member of our research team, has been involved in research that has established the utility of these STIs for measuring sexual behavior risk.73. Therefore, new infection most likely represents re-infection, not treatment failure, making this another useful marker of risk behavior. Study nurses conducted baseline clinical assessments of all participants that included: 1) antenatal care according to the South African Antenatal Care guidelines and; 2) collection of vulvo-vaginal swabs for the diagnosis of sexually transmitted infections. Swabs were refrigerated prior to transportation to a diagnostic laboratory. The BD Probetec strand displacement assay was used to detect Neisseria gonorrhoeae and Chlamydia trachomatis. Trichomonas vaginalis was detected by means of an in house PCR using the primer set SarR and SarF.

D.4.4. Random Assignment procedures: Women who have consented to participate, who have been enrolled, and who have completed the baseline assessment, including STI screening, will be individually randomly assigned to receive either standard VCT or the intervention which consists of VCT plus post-test support.  To randomize the women we will prepare random assignment cards that are placed in  sealed envelopes.  The cards will be color- coded, so blue represents the intervention arm and green represents the comparison arm.  Women themselves will be asked to select the next sequential envelope from a box.  They will then be asked to open the envelope and show the card to the person who is conducting enrollment. Randomisation is performed in permuted blocks to increase concealment of assignment from interviewers. We have successfully used these procedures for random assignment in other trials we have conducted among clients at VCT clinics.  Having participants select the cards with random assignment allocation helps to reduce concerns they may have about the randomness of allocation.  

D.4.5. HIV testing: Following the HIV pre-test counseling sessions, women who have consented will be tested for HIV according to the standard protocol used in the ANC clinic.  A participant provided a blood specimen from a finger prick for a rapid HIV test (Determine: Abbott Laboratories, Abbott Park, IL). If a reactive result was obtained with the Determine rapid HIV test, the participant's HIV status was confirmed with a second rapid test (Smart Check (WDI). In instances where a specimen was reactive to the Abbott kit, but negative on the SmartCheck, a blood specimen was sent for confirmation by an ELISA performed at the Department of Virology, University of KwaZulu-Natal. These are the tests approved and used by the South African Department of Health. Women who test negative for HIV at baseline will be offered HIV retesting once later in pregnancy and once postpartum at 14 weeks. In sub-Saharan Africa rapid tests have become the standard of care for VCT as proposed by WHO and UNAIDS (and advocated by USAID for use in developing country settings).

D.4.6. Tracing Procedures: The following systems are in place at the Clinic to maintain cohort retention and follow up: (1) A well-designed computerized follow-up program to keep track of all scheduled daily appointments and missed appointments together with contact information; (2) Study participants who miss their scheduled appointments are contacted within 24-hours by telephone if they left a telephone contact at enrollment. Approximately, 65% of ANC clients enrolled in previous studies have provided telephone contact information. This first line of contact is a reminder telephone call to the participant directly or to a close contact that is identified by the client, while maintaining confidentiality. (3) They are then given a week before a home visit is attempted if home location was provided by the clients.  Home visits are done by tracers familiar with the residential areas.  For the proposed study we will employ two full-time tracers.  If the participant has changed her address maximum information is gathered (while maintaining confidentiality of the participant) from neighbors and relatives that forms the basis of further contact. (5) A participant is considered lost to follow-up after 3 unsuccessful  attempts of telephone contact, and 2 unsuccesful home-visits.  A similar system for tracing study participants has been utilized in the previous clinical trials implemented at the nearby PMMH, and has resulted in retention rates of higher than 90%, with no adverse events associated with tracing reported.80     

D.4.7. Post Intervention Assessment: The follow-up assessment will be scheduled to coincide with women's 14 week and 9-month post-partum return visit to the clinic for the Expanded Program on Immunization (EPI).  At 14 weeks, a pap smear will be performed, STI testing will be repeated and a post-intervention behavioral survey will be conducted.  At 9 months post partum, the post-intervention behavioral survey will be repeated. The value of doing the post intervention behavioral assessment twice is that the first time period will capture the immediate intervention effect and the second time period will establish whether or not the behavior change was sustained over time. Both of the surveys will be conducted in a private room at the Umlazi Clinic.  The surveys will also be administered by an interviewer using CAPI (computer assisted personal interview). The survey interviewer will ask the questions and enter the participant's response directly onto a laptop computer. This mode of data capture improves data quality as the data will be entered on the computer one time only.  Each computer will be password protected and locked up at the medical school at the end of each working day.   

D.4.8.   Medical chart review: As women's antenatal care records cards are kept at the hospital following delivery, it is necessary for our research nurses to extract specific clinical information for individual study files to provide continuous care following delivery. The medical chart extraction form will include operational and clinical information. Operational information will include items like the length of time of each of the sessions and receipt of ARV or prophylaxis. Medical history information that will be extracted is all information that would normally be gathered during a typical ANC or post delivery visit and includes items like gestational age, pregnancy history, STI history, HIV test result, infant feeding intention, receipt of ARV prophylaxis and contraceptive use. All of the counselors, clinic and research staff, will be trained in how to log the length of the interview sessions on this form.  Medical charts extraction forms will be reviewed 4 times during the study period, shortly after the 1, 2nd and 3rd post-test counseling visits, and at the very end shortly before the 9-month post-partum assessment. This information will be collected by our research nurses and recorded on the chart extraction form. The information will then be entered into our data base by our data capturer.  

D.4.9. In-Depth Interviews with Subset of Women:  Among those women who are randomized to the intervention arm and who return for their 9-month follow-up assessment, we will interview qualitatively a subset of 20 women who have disclosed HIV test results to their sexual partners, and 20 women who have not disclosed results to her sexual partner. The purpose of the qualitative interviews is to explore in more depth women's experiences with several of the study outcomes including infant feeding, family planning, and sexual behavior change. The data from these interviews will be triangulated with data from the post-intervention surveys to gain a more complete understanding of the processes that underlie changes in behavior and decisions that women make.  These women will be systematically selected based by their response to the question on HIV serostatus disclosure from the survey interview. Survey interviewers will ask every 3rd woman who has and has not disclosed to sexual partners whether she would be willing to participate in an additional interview until we have recruited 20 women from each category for these interviews. 

In addition, we propose to document the legal education and referral component through a case study approach. We propose to do 12 case studies of women's experiences with the legal services. We will develop 4 case studies with women who seek legal services for violence, 4 case studies among women who seek legal services related to custody or maintenance and 2 case studies among women who seek legal services for death benefits and 2 case studies for women who seek legal services for development of wills. See Appendix XX for the in-depth interview guide and Appendix XX for the consent form for these case studies.  


D.4.10. Time Table: Table 1 below describes the operational time line for the proposed study. 
 
Activity	Yr 1	Yr2	Yr3	Yr 4	Yr 5	
	Q1	Q2	Q3	Q4	Q1	Q2	Q3	Q4	Q1	Q2	Q3	Q4	Q1	Q2	Q3	Q4	Q1	Q2	Q3	Q4	
Planning, consulting																					
Training ethnographers																					
Ethnographic research																					
Pilot testing and revisions																					
Operations manual develop.																					
Staff training																					
Baseline and enrollment 																					
Intervention implementation 																					
Post-intervention assess.																					
Data cleaning and analysis																					
Dissemination 																					

D.5.  Intervention implementation: The study will compare the standard model of HIV counseling to a model of HIV counseling that includes a video shown to women prior to their pre-test counseling session, an enhanced pre-and post-test counseling session, two additional post-test counseling sessions, and an active referral to post-test support groups and legal assistance.   

D.5.1. Theoretical and Conceptual Framework of Intervention: The theoretical framework for the proposed intervention expands upon Social Exchange Theory.81-85  Social Exchange Theory describes social support as a positive and reciprocal exchange.  We also draw upon Social Network literature that describes more complex positive and negative qualities of social relationships.  Social network as a structure refers to sources of both stress and support that may have a positive or negative effect on well-being and health.  Social networks can be defined by their structure and their relational content 85.  In terms of structure, the proposed intervention will focus on the dyadic relationship between the woman and her primary partner.  In terms of relational content, the intervention will address two qualities of social relations within this dyad, social support and social regulation.  First, we will focus on social support experienced by the woman from her partner.  We will measure social support in terms of emotional support (i.e. providing empathy, caring), instrumental support (i.e. providing aid in kind, money) and informational support (i.e. advice, suggestions, directives and information)81.  The second quality of the dyadic relationship between women and their partners that is addressed by this intervention is social regulation.  A social regulation perspective focuses on the constraint of an individual's behavior by another individual or societal prescription that may have either health promoting or health damaging effects85.  We know from the literature that current approaches to HIV prevention, such as promotion of condoms and monogamy, have failed many women in settings where men generally control the conditions and terms of sexual relationships86,87.  The proposed intervention is meant to enhance the quality and intensity of social support available to women in ANC in order to achieve maximum HIV prevention impact. The conceptual framework for the intervention is depicted below.  

D.5.2. Intervention Components:  The intervention is staged to handle key decisions that antenatal women need to make including: 1) Whether to test for HIV; 2) Whether to receive HIV test results; 3) Whether to enroll in PMTCT programs and accept ARV prophylaxis; 4) Whether to share HIV test results with sexual partners and encourage partner uptake of HIV VCT; 5) Decisions regarding infant feeding choices and; 6) Decisions regarding family planning choices.  The intervention is also designed to address factors that both HIV-positive and HIV-negative women face at each stage.  Prior to receiving the HIV test, women will be provided with information to better inform these key decisions through a standardized health-education video and a pre-test counseling session.  The first post-test counseling session will focus primarily on the decisions that women need to make shortly after learning their HIV serostatus and before they deliver their child, namely disclosure of HIV status to sexual partners, acceptance of ARVs, PMTCT participation and infant feeding choices.  The next two post-test counseling sessions will revisit these decisions, and will add a focus on legal education and referral, partner testing, sexual risk behavior, and family planning.  These key decisions will be reinforced through support groups to which women are actively referred. Women in the control group will receive standardized post test counseling according to the protocol recommended by WHO/CDC. Women in the control group will also receive additional post test counseling sessions; these sessions will also occur at the 6 and 10 week follow up visit and will focus on infant health.  Table 1 below compares the intervention components by study arm, and describes how they differ by HIV serostatus in the intervention arm.   The shaded text in this table indicates the counseling that will be handled by the existing clinic staff.  All of the other counseling will be handled by the counselors hired specifically for this research study.   

D.5.2.1. The video: Women who are randomized to the intervention arm will be shown a video prior to their HIV pre-test counseling session.  The objective of the video is to prepare women for the decisions that they will have to make with regard to HIV testing, collection of HIV test results, disclosure of test results to sexual partners, and participation in PMTCT programs.  The video is also meant to help prompt women to think about strategies for behavior change.  There is evidence that brief video-based interventions are cost-effective approaches that can result in significant behavior change88, and use of a video allows us to standardize a well developed presentation of health information.  Women will watch the video with other members of their pre-test counseling group.  The video will last approximately 30-minutes and will relate the stories of three pregnant women in the ANC clinic.  The video will describe the decision-making process that each woman goes through to test for HIV.  The video will also describe the decision-making process that HIV-positive women undergo to accept ARVs for PMTCT.   Two of the women featured in the video will be HIV-positive and one will be HIV-negative. All three women in the video will decide to accept HIV testing after the pre-test counseling session, and all three will return for their HIV test results.  The two HIV-positive women featured in the video will also accept the ARVs for PMTCT, however they will consider different factors in reaching this decision.  While we want to fairly represent the challenges that women face when making the decision to test and to participate in PMTCT programs, we do want to promote these two behaviors among women in the clinic and therefore the video will be designed to communicate positive messages about HIV testing and PMTCT participation.  The video will represent the benefits and potential risks of HIV serostatus disclosure to sexual partners.  Through the video the role of disclosure on women's decisions to participate in PMTCT programs, on their decisions about infant feeding choices and on their decisions about family planning choices will be stressed.  The video will highlight both HIV-positive and HIV-negative women's experiences trying to initiate sexual behavior change, in particular condom use, in their relationships. The video will be developed with the help of communication professionals from the Audio Visual Centre – HC University of KwaZulu-Natal and will be pilot tested in the ethnographic phase to ensure clarity and appropriateness of messages.  

D.5.2.2. Pre-test counseling:  The video will feed directly into the discussion that counselors initiate with the women during the pre-test counseling session.  The standard HIV pre-test counseling protocol used in the UMLAZI Section D ANC clinic consists of group counseling, conducted in the waiting room of the ANC clinic.  The group pre-test counseling is an accepted technique endorsed by WHO.  We will continue to use the group pre-test counseling model for the intervention arm, however, the counseling session will be conducted in a private room within the ANC clinic, rather than within the waiting room of the clinic. The standard model of group pre-test counseling is designed primarily to prepare women for the HIV test and provide information on PMTCT.  The pre-test counseling in the intervention arm will be more interactive, and will include more discussion with the group on the benefits and potential risks of enrolling in PMTCT programs, a discussion on disclosing HIV serostatus to male partners and information about testing sites for partners will be included.  The risk reduction component of this session is meant to prompt women to think about ways in which they may be able to initiate correct and consistent condom use with their partners to reduce their sexual risk of HIV. Counselors will encourage women to ask questions and interact with other members of the group during this session.  It is estimated that the pre-test counseling in the intervention arm will take approximately 15 minutes longer to administer than the standard HIV pre-test counseling session.  

Table 1:  Comparison of intervention components by study arm, with details on differences by  serostatus in the intervention arm.   Shaded area denotes counseling that will be done by existing clinic staff.  
	Comparison arm	Intervention arm 	
		HIV +	HIV-	
Video	
Addresses barriers to testing, disclosure, decisions about PMTCT.	Not included	  Women will not know status by this stage, therefore video will cover content relevant to both HIV+ and HIV- women	
Pre-test counseling	
Information on PMTCT	As per CDC/WHO 	Video will provide segue to the group pre-test counseling.  Group discussion will be more interactive.  Women will not know status by this stage, therefore counseling will cover content relevant to both HIV+ and HIV- women.	
Prepare women for tests	As per CDC/WHO		
Risk assessment	Not included		
Implications of results 	Not included		
1st post-test session	
Delivery of results	As per CDC/WHO 	As per CDC/WHO Guidelines	As per CDC/WHO Guidelines	
Identify sources of support	As per CDC/WHO	CDC/WHO Guidelines	CDC/WHO Guidelines	
Sexual risk reduction	As per CDC/WHO guidelines	Condom use skills, risk of re-infection; secondary prevention.	Condom use skills; behavior change w/ long-term partners.	
Information on PMTCT (infant feeding & ARVs) 	As per CDC/WHO 	As per CDC/WHO Guidelines	Not included	
Discuss disclosure plans	As per CDC/WHO 	Develop detailed plans	Develop detailed plans	
Administer structured discussion tool on violence	Not included	Used to help women assess risk of physical harm as a result of disclosure	Not included	
Mediated disclosure option provided	Not included	Women given this option	Women given this option	
Disclosure role-plays	Not included	Relevant to HIV+	Relevant to HIV-	
Partner referral for testing 	As per CDC/WHO 	As per CDC/WHO Guidelines	As per CDC/WHO Guidelines	
2nd post-test session	
	Focus on infant health.  	Revisit infant feeding, disclosure, risk reduction.  Focus on legal education and referral and partner testing.	Revisit infant feeding, disclosure, risk reduction.  Focus on legal education and referral and partner testing.	
3rd post-test session	
	Focus on infant health.  	Revisit inf. feeding, disclosure, risk reduction, partner testing and legal needs.  Add focus on family planning.  Pregnancy impact on women's health., MTCT	Revisit inf. feeding, disclosure, risk reduction, partner testing and legal needs.  Add focus on family planning.  Challenges of negotiating contraceptive use with partners.	
Post-test support groups	
Support groups offered by staff psychologist  	No active referral	31 new support group for HIV+ women	1 support group will be for all women in the intervention arm (regardless of HIV status), which will address issues all women in face in pregnancy and post-partum	

D.5.2.3. First post-test counseling session:  The standard WHO/CDC protocol for HIV post-test counseling consists of four components: (1) provision of results, which is expected to last 3-5 minutes; (2) identification of sources of support and provision of referrals, which is expected to last 4-10 minutes; (3) negotiation of disclosure and partner referral, which is expected to last 3-5 minutes and; (4) risk reduction planning, which lasts up to 5 minutes.89,90  The post-test counseling in this intervention will build on this basic framework.  At this stage of the intervention the counseling content will differ by women's serostatus.  These differences are described in Table 1. The first post-test counseling session will be tailored for the ANC setting by focusing on two decisions that women have to face immediately after learning of their HIV status, namely disclosing HIV serostatus to sexual partners and if women are HIV-positive, participation in PMTCT programs including acceptance of ARVs and infant feeding choices.  An additional 10-15 minutes of counseling time will be added for both HIV-positive and HIV-negative women to discuss issues of disclosure in more depth, and 15-20 minutes will be added for HIV-positive women to incorporate a discussion of ARVs for PMTCT and infant feeding choices to the discussion of risk reduction.   After exploring women's feelings about disclosure, counselors will use a structured discussion tool we have developed through preliminary studies to help HIV-infected women assess their risk of disclosure-related violence.  The tool is described in more detail in section D.5.2.4. Among those women who decide to disclose to their sexual partners, and for whom disclosure is a safe option, the counselor will present different disclosure approaches.   They will also talk about how disclosure may influence women's decisions about participating in the PMTCT program, her choices regarding infant feeding and her family planning choices.  Several studies that have examined patterns of HIV serostatus disclosure among women in sub-Saharan Africa have noted that women need emotional support and care of others in order to share HIV test results with their partners.30,40  One of the goals of the intervention is to establish the support necessary for women who want to safely disclose results to their sexual partners.  Role-plays will be used to build self-efficacy and communication skills for disclosure.91  

We will expand the risk reduction protocol for women in the intervention arm. Condom use skills will be emphasized in this session.  Counselors and women will talk about communication skills needed for negotiating safer sex with their partners.  This discussion will focus on the challenges that women face in initiating condom use with marital or long-term committed partners and strategies that may be appropriate for women to try to initiate these behaviors. Counselors will reinforce the benefits of using condoms consistently, and counselors will use penile models to demonstrate and practice correct condom use with women.  The standard CDC/WHO post-test counseling protocol is not tailored to address the risk reduction issues faced by pregnant, HIV-positive women.  The first post-test counseling session will include a discussion of ARVs for PMTCT and infant feeding choices.  Counselors will assess women's understandings of ARV prophylaxis for PMTCT and clarify any misunderstandings regarding efficacy, dosage and side effects.  They will also explore women's fears regarding the ARVs, and try to alleviate fears that are based on incorrect understanding of the drug effects.  Counselors will explore women's barriers to accepting ARV prophylaxis and try to develop plans to overcome these barriers that may include a plan for involving male partners.  With regard to counseling on infant feeding choices, the role of the counselors is to provide information and give encouragement and support for the feeding choices that women make.  As part of the standard practice in the ANC clinic women are counseled on safe infant feeding choices options, allowed to make an independent choice of their preferred infant feeding method, and given further information and support regarding their infant feeding choice.   Counselors currently provide information to women on the risks of HIV transmission through breastfeeding, the benefits of breastfeeding and the risks of mixed feeding.  They discuss replacement feeding options.  They help women assess the safety and sustainability of replacement feeding options including assessing women's access to safe, clean water, infant formula, utensils and other equipment required for safe preparation of replacement milk. They discuss the safe administration of replacement milk using a cup rather than a bottle.  Women's feeding choice is recorded and stamped on her ANC card. The intervention will build on this counseling by providing women with additional time to discuss the challenges they face in implementing their infant feeding choices.   The last component of this first post-test counseling session will be an active referral to post-test support groups that are facilitated by our staff psychologist, who is clinically trained (see D.5.2.7).

 D.5.2.4. A structured discussion regarding disclosure-related violence:  We want to insure that we respect women's independent choices regarding disclosure and insure that we provide women with counseling that can help minimize any negative outcomes they may face as a result of disclosure.  HIV counseling in both the intervention and the comparison arm of this trial will be client-centered, which means that the role of the counselor is to enable and support personal decisions related to disclosure.  Counselors will use a structured discussion tool to help women assess their risk for physical harm following disclosure.  We developed this tool based on the factors that were associated with disclosure-related violence in our earlier research among women in the HIV VCT clinic in Dar es Salaam.  The counseling tool consists of 5 questions that are meant to prompt discussion about the possibility of disclosure-related violence between the counselors and their clients.  The questions include: (1) Is your partner aware that you will be tested for HIV?  (2) If you told your partner you tested positive for HIV do you think he would react supportively?  (3) Are you afraid of how your partner will react if you share your HIV test results with him?  (4) Has your partner ever physically hurt you?   (5) Do you think that your partner may physically hurt you if you tell him that you have tested for HIV and your HIV test results are positive?  Counselors will support women's decisions to disclose if they answer positively or negatively to question 1, positively to question 2 and negatively to questions 3-5.  If women answer negatively to question 2, and positively to any of the following questions, 3-5, then counselors will proceed with caution and explore in more depth women's risk of disclosure related violence.  If they determine that the risk is great, they may explore alternative options including opting not to disclose, deferring disclosure to a time when women's safety can be insured or developing a plan for mediated disclosure in which the women either brings the partner to the clinic to disclose in the presence of a counselor or identifies a trusted family member or friend who can be present with the woman when she shares her HIV test results with her partner.  If women answer negatively to question 2 and positively to questions 3-5, regardless of how they answered question 1, then counselors will explore these alternative options for disclosure.   In the feasibility study we conducted in Tanzania, we trained counselors to use this counseling tool and they reported that they were able to implement it as trained without any difficulty.  

D.5.2.5. Second post-test counseling session:  Women in the intervention arm will have a second post-test counseling session that coincides with their 6-week post-partum visit to the Clinic. For many women, a single post-test session is not sufficient to address the numerous challenges that she faces having just learned her HIV serostatus.  This second post-test counseling session captures women several weeks after learning her HIV serostatus.   The opportunity for a second post-test counseling session is critical in order to review some of the important issues related to disclosure and risk reduction that the counselor and the women discussed in the first post-test counseling session, at a time when women may have overcome the initial emotional crisis associated with learning her HIV serostatus.  This counseling session will be an opportunity for HIV-positive women to talk with counselors about their infant feeding experiences and discuss any challenges that they may face in implementing their feeding choices.  This counseling session will add a focus on legal education and referral.  Counselors will introduce women to the idea of legal rights and talk about recourse options with women through the use of vignettes.  UKZN's Campus Law Clinic will work with our team to develop 5 vignettes that describe the most common legal needs o HIV+ women and women at risk for HIV and the legal options that are available to women in those situations.  In the counseling session, the counselor will read the vignette with women.  The vignettes will be a stepping stone to a discussion about the women's own legal concerns and needs. If a woman identifies a legal need, she will be referred to our partners from the Campus Law Clinic, who will be providing legal services at the clinic as a component of post test support. This session will also add a focus on partner testing.  Counselors will ask women about their partner's attitudes and experiences with HIV testing.  Counselors will describe the benefits to partner testing, and assess the barriers that women face to encouraging partner testing.  Counselors will work with women for whom disclosure is a safe alternative to develop plans for discussing testing options with their partners, including the option to test through the Umlazi Clinic.  This session will be another important opportunity to link women to the post-test support groups facilitated by our staff psychologist.

D.5.2.6 Third post-test counseling session:  The third and final post-test counseling session will coincide with women's 10-week post-partum visit to the Umlazi Section D Clinic. This visit will be another opportunity for counselors to check in with women regarding their decisions and experiences with disclosure, infant feeding, risk reduction, partner testing and legal needs.  Counselors will explore the risk reduction plans that women articulated in their first post-test counseling session and ask them about barriers that they may have faced in implementing their plans. This will be another opportunity for counselors to discuss and practice condom use skills.  This session will add a focus on family planning for both HIV-positive and HIV-negative women. The family planning counseling that will be provided to women will adhere to best practices outlined by UNAIDS92 and WHO93,94.  Helping clients make and implement an informed decision is the goal of the family planning counseling that will be provided to women.  Informed choice has two elements in family planning practice; first clients have the right to make their own choices and second clients need information and advise to help them make these choices.  The role of the counselor is to provide information, support and encouragement for women's choices regarding contraception and family planning.  Counselors will assess women's future fertility intentions, their desire for contraceptives, and the perceived barriers to utilizing contraceptives with their sexual partners.  Counselors will assess women's understanding of different contraceptive options and clarify any misunderstandings that women may have.  Counselors will introduce women to a range of contraceptive options and discuss the efficacy, ease of use, benefits and risks of each option. Counselors will spend time in this session talking and role playing the challenges women face in negotiating contraceptive use in their relationships. Counselors will spend additional time with HIV-positive women who have opted to exclusively formula feed their infants talking about the risk of pregnancy in the absence of breastfeeding.  

D.5.2.7. Post-test support groups:  Support groups will be facilitated by our staff psychologist. The purpose of these groups is to provide HIV-infected women with a safe place to share their feelings and experiences with other HIV-positive women and a place to access information or resources that they may need. Additionally, the groups will allow all women to talk about issues relating to safe sexual behaviors, pregnancy and post partum care regardless of HIV status.  The staff will facilitate one support group for HIV-positive women and one mixed group for all women (regardless of status).  The groups will meet semi-monthly. New members can join at any time, and attend as many or as few sessions as they wish.  Each session will focus on a specific topic such as disclosure, PMTCT participation, risk reduction, infant feeding and family planning.  However, the role of the facilitator will be to encourage discussion and allow new or re-emerging topics to be discussed by group members.  We will use an active referral system to refer women in the intervention arm to the support groups. VCT counselors will have a schedule of group meeting times and will be able to enroll women directly into one of the two groups.  We will use the current standard practice for these support groups among women in the comparison arm.   

D.5.2.8. Legal Services: South Africa's legal environment concerning both the rights of those infected with HIV/AIDS and the rights of women has been cited by many scholars as a potential model for other countries on the continent Csetse, J. 2003. Human Rights Watch. Policy Paralysis: A call for action on HIV/AIDS related human rights abuses against women and girls in Africa.. Despite the favourable legislation, there is a gap in application, where the majority of those infected with HIV or most at risk for HIV infection do not know that such legal protection exists. Even those individuals who know their legal rights often face barriers to enforcing them. These barriers can include issues of access in terms of availability, location and cost. In general, the legal system is largely unavailable to a majority of the population, a notion reiterated by a conference co-hosted by the AIDS Law Project in February, 2006. The group identified a number of barriers HIV infected individuals face to seeking justice, two of which include lack of information about the content of human rights as well as the inaccessibility of legal services Consensus Statement on Improving Access to legal services for people living with HIV/AIDS. Conference on HIV and access to legal services, held at WITS University February 17-18th, 2006. Retrieved January 22, 2007 from http://dedi20a.your-server.co.za/alp/images/upload/Consensus.pdf..  It is evident through multiple calls to action that increasing access to legal rights education and facilitating access to legal services for the poor are two unmet needs that could potentially affect the spread of infection as well as decrease the number of violations of HIV positive women's rights within South Africa.  We hope to respond to these calls to action through an intervention that will provide an enhanced package of HIV post test support services to women attending antenatal care (ANC) at Umlazi Section D Clinic. Legal education and services will be integrated with other HIV post test support services for women as part of this package. The primary aim of the legal component is twofold: 1) To increase pregnant women's understanding of their legal rights, and 2) To facilitate their access to legal support services 

Women in the intervention arm will be exposed to the legal education and access component at 3 different times while they are attending the clinic. 
1) Pre-test counseling:  Women who are randomized to the intervention arm will be shown a video prior to their HIV pre-test counseling session. The objective of the video is to prepare women for the decisions that they will have to make with regard to HIV testing, collection of HIV test results, disclosure of test results to sexual partners, and participation in PMTCT programs.  The video is also meant to help prompt women to think about strategies for behavior change, disclosure and negotiating risk reduction. The video will also include a brief segment on women's legal rights to begin to raise awareness among women of their legal rights and possible legal recourse for issues that they may face.   
2)  Post-test counseling:  Women in the intervention arm will have a second post-test counseling session coinciding with their 6-week post-partum visit. This counseling session will have a legal focus. Research Nurses will introduce women to the idea of legal rights and talk about recourse options with women through the use of vignettes. The vignettes will be a stepping stone to a discussion about the women's own legal concerns and needs. 
3) Legal services:  Research Nurses will actively refer women who describe legal concerns to the legal staff from the University's Campus Law Clinic. These legal staff will be available on site to meet with women who are referred by the Research Nurses. The staff will comprise of law students, a candidate attorney and a lawyer and will follow a similar model to existing Campus Law Clinics. Appointments to see the clinic staff will be coordinated through the Research Nurses on the project. These services will be provided to the women free of charge, provided each woman in the intervention arm meets the eligibility criteria set by the Campus Law Clinic. 

Training of Research Nurses – We propose that the law faculty will develop and facilitate an initial training workshop for our Research Nurses in Year 1 and a shorter booster training for the Research Nurses in Year 2.  The objectives of this training will be to: 
o	Teach the Research Nurses about legal rights and legal services available in KwaZulu Natal 
o	Teach the Research Nurses how to educate female ANC patients at the Umlazi Clinic about their legal rights using specific vignettes 
o	Train the Research Nurses how to refer female patients who have legal needs to the Law Clinic staff. 
This training will be modeled after other trainings that have been designed and delivered by Street Law. The scope of the legal training will be defined jointly by the faculty from Law and Medicine prior to the development of the training workshop. Based on our preliminary assessment of legal needs of women in Umlazi it is likely that the topics that will be covered in the legal training include: protection orders for domestic violence, maintenance, custody, divorce, and development of wills. In addition to the 3-day training to be held prior to the launch of the intervention, we request assistance with a shorter booster training session for the Research Nurses during the second year of the intervention.   

Material development – We propose working with the Law faculty to develop vignettes that describe the most common legal issues faced by HIV+ women and women at risk for HIV and the legal options that are available to women in those situations. The research team will develop these vignettes based on formative research; feedback will be solicited from the Law faculty before the vignettes are used. These vignettes will be presented to the Research Nurses during the training and then implemented by the Research Nurse in the 6-week post-partum counseling session .We also propose that the Law faculty will help us identify and adapt educational materials (such as pamphlets about legal rights) that Research Nurses can use when talking with female ANC patients about their legal needs. 

Providing the Legal Services –We propose that the UKZN Campus Law Clinic provide the legal services at Umlazi Section D Clinic for women in the intervention arm of our trial. As discussed in the training section, we will define the scope of the services that will be provided by the law staff in Umlazi prior to the launch of the intervention. Based on our preliminary assessment of the legal needs of women in Umlazi it is likely that the scope of the services will include maintenance, protection orders for domestic violence, custody, divorce and development of wills. The UKZN Law Clinic staff will follow service delivery procedures that are similar to those that they utilize in their main office at Howard Campus. We will work with the Lawyer in charge of the Campus Law Clinic to modify the data records that they maintain for their clients to ensure that we standardize it with other data collection for our trial.   

Monitoring and Evaluation - This study represents an ideal opportunity for the faculties of Medicine and Law to collaborate on a comprehensive intervention that can be rigorously evaluated through a randomized controlled trial design. We firmly believe that integrating services will result in better health outcomes for women, and we propose to document the impact of these comprehensive services for women through this intervention trial. We describe below some of the benefits we foresee to this collaborative partnership between the Faculty of Medicine and Law.
(1)  Documenting women's legal needs: 
o	Our pre and post evaluation assessment will allow us to measure knowledge of legal rights, knowledge of legal recourse, perceived legal need, and experiences of discrimination of the 1,495 women who will participate in our study. 
o	We anticipate that the baseline assessment will yield strong empirical evidence documenting current levels of legal awareness and need within a large sample of individuals in the province. 
(2) Evaluating the impact of providing legal education and services: 
o	We will measure the impact of the legal education component by comparing women's scores on legal questions on both the baseline and follow up measure. We will do this for each of the legal questions that are asked in the baseline survey. In this way, we will be able to evaluate if the legal component of the program increased women's understandings of their own rights.
o	We propose to document how legal services impact women through a case study approach. The case study approach that we are proposing will complement the quantitative data. We will select and follow prospectively women who have received legal services through the intervention to describe in depth how they utilized the legal services and what impact it had on them.  
(3) Describing the process of providing legal services. 
Through the process evaluation component of our intervention trial we will also be able to document the following: 
o	the number of women given legal referrals by our Research Nurses
o	the number of women meeting with law staff at UKZN clinic
o	the number of women seeking legal recourse 


D.6. Feasibility of the intervention:  When designing the experimental intervention to be tested in the proposed study we were sensitive to the fact that there is currently a standard protocol for HIV VCT that has been endorsed by WHO and CDC and is widely accepted and utilized in the field.  Our intervention builds on the strengths of these existing protocols.  The need to create a feasible intervention must be balanced with the need to create an intervention that is intense enough to have an impact.  We have designed a model of counseling and post-test support that does not require complex reorganization of VCT and PMTCT services.  The video will be shown to women as part of their group HIV-pretest counseling session.  We will extend the length of the pre-test counseling session by about 30 minutes to cover issues related to disclosure and PMTCT participation.  The first post-test session will be lengthened by an additional 20-30 minutes for both HIV-positive and HIV-negative clients.  There will be two additional 30-45 minute post-test counseling sessions for women that coincide with their 6-week and 10-week post-partum visits.  We designed the intervention to take advantage of the fact that women are already returning to the Family Clinic at regular intervals for the immunization of their children.  Finally, HIV-infected women will have the option to enroll in post-test support groups facilitated by our staff psychologist..  We feel that in order to effectively counsel women through all of the issues that they face when learning of their HIV status, this additional counseling time and post-test support is warranted.

  
D.7. Comparison Condition:  The comparison arm is designed to provide standard of care for HIV VCT.  Currently, the VCT that has been provided by Umlazi Section D ANC since 2001 is in accordance with the National VCT guidelines established by the South African Department of Health.  These national standards are based on the international standards established by WHO and CDC and also take into account the cultural context of South Africa. There are four full-time HIV counselors and five professional nurse counselors in the clinic.  All counselors have received training in HIV VCT as per the South African national standards.  Women in the comparison arm will meet with research counselors at the 6 and 10-week postpartum visits.  To minimize any bias that may be associated with contact time with counselors, the 6 and 10-week counseling sessions for women in the comparison arm will be comparable to the length of sessions for women in the intervention arm.  This will provide women with an opportunity to talk about concerns that they may have about their health of the health of their infant.  In their introductory remarks, the counselors will indicate that the primary focus of the session is on infant health.  Because these sessions, like all others, will be client-centered, it is possible that women in the comparison arm will raise questions that are similar to the issues being discussed with women in the intervention arm, namely disclosure, partner testing, and family planning.   Counselors have an ethical obligation to respond to these issues when raised by the women.  The counselors will use skills that are part of the standard skill set of counselors at the clinic, including actively listening to women's concern's, responding empathetically, and offering referral information when appropriate.   The counseling tools that we use for women in the intervention arm, for example the disclosure role-play scenarios, will not be used when counseling women in the comparison arm on disclosure.     
  
D.8.Behavioural and clinical Assessments:  The survey instrument will be developed following standard practices.  The items will be written in Zulu, pre-tested on a few respondents, exit interviews will be conducted to determine if the content was understood as expressed, changes made, and then the survey will be translated into English by an independent translator to determine whether the content remains as planned.  Alterations will then be made, back-translated into Zulu and then pre-testing will be conducted a second time, with approximately 20 representative respondents.  Scales and variables that will be used to measure the key study outcomes such as emotional distress, social support, MTCT acceptance, and partner uptake of VCT will go through another version of refining and re-pilot testing with an additional 20 women. See Appendix 11 for a table of constructs, variables and measures to be used in the survey assessments.  The instrument development will take place during the phase of ethnographic research.  

D.8.1. Primary Outcomes
D.8.1.1. STI Incidence:  Incidence of STIs will be measured and compared among women in the intervention and control arms. Women are screened for a range of STIs upon enrollment to the ANC clinic including (Neisseria gonorrhea, Trichomonas vaginalis, Syphilis, and Chlamydia trachomatis) at baseline.  All women who test positive for any STI are treated immediately.   For the purpose of our study we will measure incidence of Neisseria gonorrhea, Trichomonas vaginalis, and Chlamydia.  These three organisms offer advantages in the evaluation of behavior change interventions in that they are curable with a single-dose treatment regimen and they are sufficiently prevalent in the target population to warrant inclusion.101  All women who return for the follow-up visit 14 weeks post-partum will be re-screened for Neisseria gonorrhea, Trichomonas vaginalis and Chlamydia. All women in the intervention and control arms who are screened for STIs at 14 weeks, will also have a PAP smear done at the time of the cervico-vaginal examination.  Women found to have an STI at this visit will be treated at that visit.   We feel it is important to include a biological outcome as one of our primary study outcomes to measure intervention efficacy. This will enable us to triangulate our findings from self-reported risk behavior data we gather from the women to gain a more complete understanding of behavior change.72  

D.8.1.2.  Correct Condom Use:  The consensus recently has been to include measures of both correct and consistent condom use for a more robust behavior change evaluation.72 To measure correct condom use we will ask women to recall during the last sexual encounter in which they used condoms whether they and/or their partner performed the following discrete steps:  1) checked the condom's expiration date; 2) whether their partner had inserted his penis prior to putting on a condom; 3) whether they left space at the tip of the condom; 4) removed air from the tip of the condom;  and 5) whether they or their partner held the rim of the condom on withdrawal. We will calculate the proportion of women who report that they performed all five steps correctly.   

D.8.1.3.  Consistent Condom Use:  Consistent condom use has been selected as a primary outcome based on demonstrated effectiveness in reducing sexually transmitted HIV infection.102,103 104  To measure consistent condom use we will use the Partner grid included in Appendix 12.  In completing this grid, we will ask women about each of their sexual partners, how many times they have engaged in sex with these partners in the last 30-days and how many of these times they used a condom. We will calculate the number of unprotected sex acts by subtracting the number of times a condom was used from the number of sex acts.  

D.8.1.4 Partner uptake of HIV VCT: We will monitor the demand for HIV testing by partners of women in both arms of the study.  We will focus on the primary partner of women, as defined by a partner that they have been with for at least 6-months.  This is a definition that we have used in previous studies and one that we feel will capture the majority of stable primary partnerships in this population of women.  At baseline we will ask all women whether their partner has ever tested for HIV. At the post-intervention assessment we will ask women whether their partner has been tested for HIV since their enrollment in the study.  We are interested in partner uptake of HIV testing at any VCT clinic, not exclusively within the Family clinic.  We recognize that the most accurate rate of HIV VCT uptake by partners would be achieved by asking the partners directly. However, we do not want to compromise the safety and confidentiality of women by contacting their partners for interviews.

D. 8.1.5:  Acceptance of HIV VCT: The clinic nurses indicate in every woman's file whether HIV testing was accepted or declined.  We will end up enrolling and collecting baseline data on some women who opt not to test for HIV. Women who are enrolled but decline HIV testing will receive ongoing antenatal care and offered the opportunity to test for HIV at subsequent visits. The data that we have from the baseline interview will allow us to compare demographic and risk characteristics among women who accept HIV VCT with women who decline HIV testing. We will also be able to measure the effect of the intervention on women who initially refuse to test for HIV at baseline, by comparing HIV testing outcomes in the intervention and control arms.

D.8.1.6. Receipt of ARV Prophylaxis:  We will measure receipt of ARVs by asking women at the 14 weeks post-partum follow-up assessment whether they received the dose of NVP offered through the ANC clinic.  The clinic keeps records in every woman's file on whether she has received NVP.  We will compare the self-reported rates of acceptance from our survey with the rates that are recorded in the clinic records.  

D.8.1.7. Adherence to national infant feeding guidelines: We will measure infant feeding choices by asking women to recall whether in the first six months post-partum they practiced exclusive breastfeeding, exclusive replacement feeding or mixed feeding.  Reports of either exclusive breastfeeding or exclusive formula feeding will be considered in adherence with national guidelines. Reports of mixed feeding will be considered non-adherence to national guidelines. 
  
D.8.1.8. Family planning use:  At the 14 week and 9-month follow-up assessment we will ask women whether they are currently using any contraceptive method.  We will calculate the proportion of women who are currently using any contraceptive method including female sterilization (performed in the past 14 weeks or 9-months), pill, IUCD, injectables, male condom, female condom, or periodic abstinence, withdrawal, emergency contraception.  

D.8.1.9 Family planning intention:   Among women who answer that they are not currently using any method, we will ask them whether they intend to use a contraceptive method to delay or avoid having a pregnancy in the next 12-months.  We will calculate the proportion of women who indicate that they intend to use a contraceptive method in the next 12-months.  This measure of intention to use contraceptives is commonly used in Demographic and Health Surveys105.    
   
D.8.1.10.   Perceived Social Support:  The focus of the intervention is the social support exchanged between the HIV infected woman and her partner.  Serovich found that perceived social support rather than actual social support was correlated with mental health106.  We will use a modified version of the Social support index developed by Wilcox107,108.  The 18-item scale measures perceived emotional, tangible and informational support for an index person, which in the case of this study will be the woman's partner.  A score is derived by summing the number of items responded to in the 'support present' direction.  

D.8.1.11.   Emotional distress:  We will measure symptoms of emotional distress through the Hopkins Symptom Checklist-25 (HSCL-25).  This screen has been validated among HIV positive pregnant women in sub-Saharan Africa109.  The HSCL-25 demonstrated adequate internal consistency (alpha 0.93) among women in ANC.  See a copy of the scale in the Appendix 14.  

D.8.1.12. Partner Violence: From our previous research, we found that using specific behavioral events rather than a cover term such as 'physical violence' is more useful for the measurement of violence.  This has been corroborated by violence research from other settings, including South Africa47,110  We will measure sexual and physical violence using an adaptation of the Conflict-Tactic Scale111.  (See Appendix 13)  We adapted this scale for use in the research that we conducted in Tanzania and the scale demonstrated good internal consistency and reliability for physical and verbal abuse in this setting60 .  The modified scale demonstrated good reliability and internal consistency in the eight countries where WHO conducted a study on women's health and gender-based violence and in a South African study conducted by Jewkes112,47.  At baseline we will ask women to recall their experiences with violence during pregnancy and prior to pregnancy. At the post-intervention assessment we measure women's experiences with violence in the 9-month period of time since testing.  We will adhere to WHO ethical and safety guidelines for conducting research on violence against women.  We will have a referral list of social and legal services available to women who report current  violence113.

D.8.2. Secondary Outcomes
D.8.2.1. Length of time for counseling sessions:  Counselors will maintain a counseling log form for every woman in the intervention and the comparison arm.  Among other things reported on this log form, counselors will report the beginning and ending time of each counseling session.  

D.8.2.2 Ongoing counseling: We will monitor the ongoing use of counseling services by women in both arms.  Counselors will record every counseling session that is conducted with clients on the counseling log forms.  Through these counseling logs forms we will also track the number of women who return for counseling with her partner. We will start a separate counseling log for those partners who return to the clinic either on their own for HIV counseling or with their partner.  

D.8.2.3. Quality of counseling as perceived by the woman: We will ask 5% of women randomly sampled using exit interviews after their counseling sessions about their perception of the counseling quality that they received.  They will be asked to evaluate the following skills of the counselors viz.  listening, responding with concern and support, supporting decisions and making appropriate referrals using a five-point scale, one being weak and five being strong. In addition, at the 14 week and 9 month assessment, we will ask about their perceived quality of care.   


D.8.2.4. Costs of the standard VCT and the VCT plus post-test support: We will gather information on costs of the intervention from the clinic and the client's perspectives.  We will quantify costs of each element of the counseling and testing process. This will be done through financial records from the clinic.  We will ask clients on the 9-month follow-up survey about costs they may have incurred accessing the services including transportation costs and wages lost during the period of time participants were in the clinic.  

D.8.3. Independent/Predictor/Mediating Measures
D.8.3.1. HIV Serostatus:  We will gather information on women's HIV serostatus from the ANC records.  We have powered the study to be able to stratify all of our analyses by serostatus.  

D.8.3.2. Demographic:  Basic demographic characteristics of participants including age, marital status, educational level, and socioeconomic status will be gathered.  Socioeconomic status will be measured by indicators such as type of roofing and flooring, type of toilet facilities and ownership of radios and televisions.  

D.8.3.3. Parity:  We will ask women about the number of pregnancies that they have had in the past and the number of children who are currently living.  For women who report that they have children we will ask whether any of the children are HIV-infected.  

D.8.3.4. Disclosure to sexual partners: To measure the rates of HIV serostatus disclosure to sexual partners we will ask women who return for the survey interview 9-months post-partum whether they have shared their HIV test results with their primary sexual partner.  

D. 8.3.5. Duration of time since testing:  We will assess the gestational age at which women received testing and the gestational age at delivery to calculate the duration of time between testing and post-intervention assessment.  This information can be gathered from clinic records.  

D.9. SAMPLE SIZE 

D.9.1. Aim 1: Our first aim is to describe the demographic, social and psychological factors that affect sexual risk behaviors, ARV uptake, infant feeding choices, partner testing, family planning intentions and HIV serostatus disclosure to sexual partners.  To describe these factors we will conduct 64 in-depth interviews with women at 6-weeks post partum, and 16 in-depth interviews with male partners of ANC clients. Because we are hypothesizing that disclosure plays a role in women's decisions regarding PMTCT participation, infant feeding and family planning, we will stratify our qualitative sample by disclosure status.  We will sample 10 women who have disclosed their status to their partner (5 HIV-positive, 5 HIV-negative) and 10 women who have not disclosed to their partner (5 HIV-positive, 5 HIV-negative). The particular sampling strategy that we will use is criterion sampling, in which we identify women who represent these pre-determined criteria.114  

D.9.2. Aim 2:  We have conducted power analysis for each primary study outcome.  We have determined that reduction in STI incidence is the factor driving the sample size for the study.  Table 3 below shows the sample size required to detect significant differences in STI incidence across the two arms.  The criterion for significance (alpha) for all power analyses has been set at 0.05.  The tests are 2-tailed, which means that an effect in either direction can be detected and interpreted.   We hypothesize that the intervention will lead to lower rates of STIs. Prevalence data on STIs among pregnant women from KwaZulu-Natal indicate that 52% of women had at least one STI, 12.9% were diagnosed with Chlamydia, 7.8% were diagnosed with Neisseria gonorrhea, and 23% were diagnosed with Trichomonas vaginalis8.  Based on earlier research our team conducted in Durban we established the incident rate of gonorrhea among a periurban sample of ANC attendees in 2000 was 23%.  We have estimated a 22% annual incidence rate of either Neisseria gonorrhea, Trichomonas vaginalis or Chlamydia among HIV-positive women and 18% among HIV-negative women.    Results shown in Table 3 indicate that a sample size of 279 HIV-positive women and 295 HIV-negative women will be adequate to detect a minimum of a 9% difference in rate incident STIs among HIV-positive and an 8% difference among HIV-negative women in the intervention and control arms at the 9-month follow-up assessment.  We feel that this is the minimum level of change that would be meaningful from a policy and program perspective.

Table 3:  Required sample size per arm to detect a difference in STI incidence
STI incidence (base rate)	Effect size (% point change) and Required total sample size per arm	
	.08	.09	.10	
HIV+ women (.22) 	361	279	221	
HIV- women (.18) 	295	226	177	

D.9.3. Summary of sample size calculations: A sample size of 295 HIV-negative women and 279 HIV-positive women per arm will enables us to answer all primary research questions, and will enable us to stratify all of our analyses by serostatus.  Table 4 describes the detectable effect sizes that we will have for each primary outcome with this sample size.  We will have adequate power to detect a minimum difference of 11% for our most difficult test of intervention effectiveness (partner violence among HIV-positive women), and comparably low differences in other major study aims.  The power calculations were conducted using a normal approximation with SPSS Sample Power program.  We expect attrition to vary by study arm from pre-test to post-test (with higher rate of return for HIV test results in the intervention arm) because this is one of our primary study hypotheses.  After this, we expect the attrition across study arms to be consistent.  The attrition rate among women who have returned for HIV test results is expected to exceed 90% in both arms.  We will need to enroll 1,495 women in the trial to insure we have adequate women at the 9-month assessment to measure the study outcomes.  


  Table 4:  Summary of detectable effect sizes with 1,495 participants 
Outcome	Population	% change	% point difference 	
HIV serostatus disclosure to sexual partner115	HIV+ 	.25-.36	11%	
	HIV-	.50-.62	12%	
Partner uptake of testing116 	HIV+ 	.25-.36	11%	
Consistent condom use117 	HIV+ 	.21-.32	12%	
	HIV-	.30-.41	11%	
Correct condom use118 	HIV+	.60- .71	11%	
	HIV-	.40-.51	11%	
Acceptance of VCT3	HIV+	.78-.87	9%	
	HIV-	.82-.90	8%	
Receipt of ARVs3	HIV+	.58-.69	11%	
Adherence to infant feeding guidelines119	HIV+	.80-89	9%	
Contraceptive intention50	HIV+	.34-46	12%	
	HIV-	.27-.38	11%	
Perceived social support38 	HIV+ 	.24-.35	11%	
	HIV- 	.26-37	11%	
Emotional distress106 	HIV+	.40-.28	12%	
	HIV-	.15-.07	8%	
Physical violence in past 9-months120	HIV+	.40-.29	11%	
	HIV-	.34-.24	10%	


D.11.  Statistical Data analysis:
  
The primary purpose of the quantitative data analysis is to conduct a stratified analysis comparing HIV-positive and HIV-negative women randomized to the standard versus the enhanced counseling on the primary study outcomes described in Aim 2.  We have the following primary hypotheses to test intervention effect among women randomized to the intervention as compared to women randomized to the comparison arm at the 9-month post-intervention assessment: (H1) There will be a significant decrease in sexual risk of HIV as measured by STI incidence, correct and consistent condom use and partner testing.  (H2) There will be a significant decrease in mother to child transmission risk as measured by increased uptake of HIV VCT, increased uptake of ARVs, increased adherence to national infant feeding guidelines and increased contraceptive uptake. (3) There will be a significant improvement in psychosocial outcomes associated with PMTCT participation as measured by an increase in perceived social support, and a decrease in emotional distress and partner violence.  Statistics used to test these hypotheses will include comparisons of outcome variables across independent variables using contingency table analysis (log linear Poisson models) with Chi-square tests for significance, t-tests, and F-test for appropriate bivariate differences and multivariable analysis (ordinary least squares multiple regression for continuous outcome variables, and logistic regression for dichotomous outcome variables).  Diagnostic tests for multicollinearity will be conducted prior to multivariable analysis.  All analyses will be conducted separately for HIV-positive and HIV-negative women, given that the baseline rates vary between the two groups and expected change from pre to post-intervention assessment also varies by HIV serostatus.  We will examine how HIV serostatus disclosure to partners influences the effect of the intervention on each study outcome by including HIV serostatus disclosure as a covariate in our analyses. To compare operational outcomes across study arms (Aim 3) we will use contingency table analysis with Chi-square tests for significance and t-tests and F-tests for appropriate bivariate differences.   

D.12. Qualitative data analysis:  

All in-depth interviews and FGDs will be audiotaped, transcribed, translated, coded and computerized for analysis.  Analysis of textual data will consist of two techniques: (i) the identification of recurrent patterns and themes:  Qualitative data analysis is the search for patterns in data and for ideas that help explain the presence of those patterns121.  Textual data will be carefully read to identify recurrent themes, and to obtain familiarity with complex interaction between men and women.  Codes will be created and assigned to specific sections of text so that the text can be easily and meaningfully searched122. After the coding scheme has been finalized, the ethnographers will be trained to apply the codes to the transcripts.  To ensure inter-coder reliability, 50% of data will be double-coded.  Coded text can be later extracted from each of the interviews so that a more domain-specific reading can be undertaken. We will import the translated, coded and computerized interviews and focus group discussion transcripts into Atlas ti, a qualitative analysis software package.  We will use Atlas ti to help identify recurring themes, concepts and terms that are relevant to the analysis.   (ii) Use of domain matrices:  Matrices and tables that categorize and display data will be used to help the reader quickly understand the dimensions by which the data are categorized and facilitate comparisons.  Matrices will help to display the full, yet condensed data set122. For example, a matrix may be developed to describe the decision-making process that women go through to discuss testing with their partners.  
 
D. Data management AND QUALITY CONTROL

D.13.1 Data management: Data will be primarily managed on-site by a data entry and management team.  
D.13.1.1  Survey data:  
Data will be collected through Computer Assisted Personal Interviews (CAPI).  We will pilot our questionnaires for the baseline survey in 2 different ways. Firstly, we will conduct cognitive interviews with 5 women to evaluate our baseline survey. During these interviews, we will ask each woman the questions within each section of the baseline survey. At the end of each section, we will ask each woman a series of questions about the content of the section in order to assess their understanding of each question, to determine whether the response categories are sufficient, and to evaluate whether questions need to be added, deleted or altered. Following the cognitive interviews, we will make any necessary changes to the baseline survey instrument. We will then pilot the baseline survey in its entirety with 15 women from the Umlazi Section D clinic. 

D.13.1.2 Qualitative Data: 
Ethnographers will conduct the in-depth interviews in Zulu.   Field guides for the semi-structured in-depth interviews and focus group discussions will be translated into Zulu and back translated into English prior to use. All interviews will be audiotaped.  Ethnographers will be responsible for transcribing each interview.  We will employ a translator who will translate the Zulu transcripts into English.  The data entry team will then computerize these English transcripts for analysis purposes.  The data manager will supervise staff, and there will be regular quality assurance checks conducted to assure consistency in methods of translation and transcription. The qualitative data files will be backed up on a daily basis on all project hard drives, the South African Co-PIs' hard drive and on CD-ROM.  Qualitative interviews will be randomly checked for content, accurate transcription and translation.  

D.13.2.  MONITORING QUALITY CONTROL: 
D.13.2.1. Protocol compliance
a.	The Project Coordinator will be responsible for auditing 100% of the informed consents to ensure that consent for participation in the study was obtained according to Good Clinical Practice (GCP). 
b.	Counseling Observations: The staff psychologist will be responsible for observing 5% of the patient's visits at baseline, 6 weeks and 10 weeks. These sessions will be randomly sampled. The staff psychologist will have a checklist to verify that the content of the session adheres to the protocol. The psychologist will also evaluate the quality of the nurse's counseling. These evaluations will be used to maintain quality of care in delivery of services across both the control and intervention arms. 
c.	Exit Interviews: The survey interviewers will be responsible for conducting exit interviews with 5% of the participants following the baseline, 6 week and 10 week sessions. These sessions will be randomly sampled. These interviews will address perceived quality of services received and they will be used to maintain quality of care or improve quality of care as needed across both the control and intervention arms. 

D13.2.2 Quality Control IN DATA MANAGEMENT
a.	The Project Coordinator will be responsible for auditing 5% of the patient's medical charts at the baseline, 6 week and 10 week visit for protocol compliance. These charts will be randomly selected.  The audit will involve a comparison between the woman's antenatal card and the infant's road to health card with our Medical Extraction Form. The nurses will extract a number of pieces of valuable information from these cards onto our Medical Extraction Form . We want to ensure that the nurses are reliably extracting information from these two sources into our Medical Extraction Form.
b.	The Project Coordinator will also be responsible for validating 5% of data captured by the data capturer.  She will verify data captured on the database against the Medical Extraction Forms.


D.14. Data Safety and Monitoring Plan: 
 Our data safety and monitoring plan includes the establishment of a Data Safety and Monitoring Board (DSMB).  The DSMB will be responsible for general oversight of the study including reviewing study protocols, monitoring project implementation including accrual and drop out rates, reviewing interim analyses of outcome data, and monitoring adverse events and human subjects issues.  The DSMB will establish clear stopping rules for the trial if adverse events are deemed to reach an unacceptable level, if drop out rates across study arms are unacceptably different, if interim analysis indicates that the intervention effect is significant enough to warrant that all women in the trial be given access to the intervention, or if it becomes apparent that the trial cannot be concluded successfully. Interim analyses will be planned each year during the intervention implementation phase.  The primary risk to participants in this trial is the potential social harms that may be associated with HIV diagnosis.  We will ask study participants to return to the research site as well as provide participants with a card containing information on how to contact the research staff to report such incidents as HIV-related family disruption, acts of discrimination and physical harm.   Research staff in both arms of the study will be trained to complete descriptions of adverse events that will then be sent electronically to the U.S. PI and the South African Co-PI's.  All adverse events and serious adverse events associated with the procedures will be appropriately reported to the JHU Committee on Human Research, the University of KwaZulu-Natal IRB and the DSMB.  We define adverse events as any undesirable, unintended reaction or event (whether expected or unexpected) that results from study procedures or study interventions.  We define serious adverse events as any subset of adverse events that are fatal, life threatening, require hospitalization, or prolong existing hospitalization or result in persistent or significant disability.  No serious adverse events are expected with study activities. Adverse events and serious adverse events will be reported to IRBs within the required time frame that each IRB specifies.  The time frame for reporting to the IRB at the University of KwaZulu-Natal is within 7 days, and to the JHU IRB we are required to report the event within 10 days of being detected. We propose reporting adverse events to the DSMB within 10 days of an adverse event coming to the attention of a PI and within 5 days for serious adverse events.
 

D.15. Project Management:  

The project will be managed jointly by the PI, Dr. Suzanne Maman, and the Co-PI Dr. Dhayendre Moodley.  Dr. Maman will be responsible for overall quality of the study, adherence to the study protocol, data management, analysis of study data, scientific integrity of the study results, interpretation of the study results and dissemination of the study findings.  Dr. Maman will also oversee the overall financial management of the project and will coordinate the transfer, cleaning and quality control of the study data.  Dr. Moodley will be responsible for management of the South Africa-based staff, oversee the scientific aspects of the study in South Africa and the financial management of the South Africa-based activities.  Quarterly site visits by either Dr. Maman or the U.S. Study Coordinator will be arranged.  Regular bi-monthly conference calls will be scheduled with Dr. Moodley to assure that monitoring of activities is maintained.  We will hire two Study Coordinators for the project, one based in North Carolina and one based in South Africa.  The responsibilities of these two study coordinators will be to backstop the PI and the Co-PI's to ensure the overall quality of the study.  


D16.  Staff Training:
 
All project staff will undergo extensive training on the goals and study procedures, as well as in-depth training on specific duties and responsibilities.  We will develop a manual of operations that will be regularly updated and reviewed with the staff.  We will conduct an annual staff retreat in which we will refresh staff on the study procedures.  The different categories of staff will undergo specific training related to their responsibilities.  The counselors will be trained by Dr. Kagee (co-investigator) a trained clinical psychologist specializing in HIV related counseling.  The counselors will build counseling skills through role-plays with clients. Prior to study implementation each counselor must conduct a 'certification' counseling session.  Based on satisfactory scores counselors will be 'certified' to begin study implementation.  Every year counselors will receive a refresher course and will be required to get re-certified.  


D17.  Limitations: 

As in most prospective studies, this study is limited by selection bias.  However, the study's timeframe is not long enough to raise significant concerns over uncontrolled temporal effects.  The Umlazi SED ANC clinic has considerable experience in following up individuals for extended periods of time and has demonstrated in previous studies that loss to follow-up can be kept to about 10% per year.  It is also possible that women who are most likely to experience difficulty disclosing results to their sexual partner and who may be most likely to suffer from negative outcomes as a result of disclosure, will choose not to participate in the study.   Since we will randomize women to either the intervention or comparison arm, this bias should be evenly distributed between arms, and therefore we will be able to still determine the relative effect of the intervention in this population of women. 


E.  Human Subjects 

Research ethics experience of the study team:  The research team has considerable experience with research ethics.  The PI, Dr. Suzanne Maman, works extensively with the Johns Hopkins University Bioethics Institute, as the Assistant Director of a fellowship program in International Research Ethics for scientists from sub-Saharan Africa.  She has been involved with facilitating short-course ethics training in Malawi, Ethiopia, and Tanzania.  She is also involved in an empirical study designed to describe research participant understanding in HIV prevention studies in South Africa, Malawi, and Haiti.  All members of the study team have participated in NIH-funded studies in the past and have successfully completed human subjects training (certifications are attached). 

Voluntary choice regarding disclosure: We recognize the potential risks associated with HIV serostatus disclosure to sexual partners among women.  We have conducted prior research studies on the associations between HIV and violence against women and we have reviewed the literature extensively to further understand the positive and negative outcomes of disclosure for women.  There is some evidence to suggest that women who disclose HIV test results to their partners are at risk for experiencing negative reactions, including physical violence.  While the rates of HIV serostatus disclosure related violence are relatively low in the U.S. (between 0.5-4%) the risks of violence following disclosure among women in sub-Saharan Africa is higher (3.5%-14.6%).  The potential risks associated with disclosure have to be weighed against the possible individual and public health benefits of disclosure.  Expanded awareness of risk that comes with disclosure may enable couples to make informed choices about behavior change and reproduction that may ultimately lower the incidence of HIV.  There are also risks associated with not disclosing HIV test results with a sexual partner, including the risk of transmission to a partner and anxiety associated with concealing this information from a partner.  We have developed a counseling approach that is designed to help women more carefully weigh the risks and benefits of disclosure.   Through the intervention that we are testing in this proposal women will spend more time with the counselors discussing disclosure.  The HIV counseling will be client-centered, which means that the role of the counselor is to enable the client to make independent decisions related to disclosure.  Counselors will use a structured discussion counseling tool to help women assess their risk for physical harm if they disclose HIV positive test results to their partner.  We developed this tool based on the factors that were associated with disclosure-related violence in our earlier research among women in the HIV VCT clinic in Dar es Salaam.  The details of this screening tool have been described in section D.5.2.4.  

Value to Host Country:  This protocol responds to needs identified in the host country.  The study builds on capacity and has the potential to be of lasting value to host countries.  A report commissioned by the South African Department of Health provides an assessment of the national pilot PMTCT program1.  Among some of the recommendations made in the report it was stressed that there is a need to “provide on-going psychological and emotional support to HIV positive women as well as advice on disclosure.”  The proposed intervention directly addresses the social and behavioral challenges of implementing PMTCT interventions that have been identified in the South African Department of Health report.  

Voluntary informed consent:  The research site at Umlazi D Clinic in Durban, South Africa has extensive prior experience obtaining informed consent from women for clinical trials within this cultural context.  The informed consent procedures for this study have been designed to maximize understanding of potential risks.  All consent forms will be translated into Zulu and back-translated into English to ensure correct use of language.  Consent forms will be read aloud to participants by study interviewers.  After reading the consent forms prior to seeing a signature, interviewers will ask participants to summarize the study and explain the reasons why they want to participate.  At this point any misunderstandings regarding procedures, risk or benefits can be clarified.  If there are cultural, literacy or political reasons why a signature is not appropriate, individuals will be allowed to mark the consent form with an 'X'.  Separate informed consent will be obtained for each time that data are collected including the preliminary studies, the baseline survey, in-depth interviews with selected survey participants, exit interviews from specific counseling sessions, observation of specific counseling sessions and the 9-month post-partum follow-up survey.  Women will be informed during the consent process that their clinic charts will be reviewed by the study staff, and some data will be extracted, including information on their HIV testing decision, whether or not they received ARV prophylaxis for PMTCT, and the length of their counseling sessions.  

Confidentiality:  Successful implementation of the trial will require that strict confidentiality of all study participants be maintained.  Rapid HIV testing will be anonymous to afford the highest level of confidentiality.  For the survey and qualitative data collected as part of the study, participants will be assigned a unique identification number.  All data and information collected will be kept in locked filing cabinets.  Counselors, interviewers, data entry clerks, data manager and tracers will all be trained on procedures to maintain confidentiality and will be required to sign an oath of confidentiality prior to study implementation.  We will ask participants to return to the research site as well as provide them with a card that has contact information for research staff to report such incidents as HIV-related disruption of family, as well as physical and social harms.  This will enable research staff to make appropriate referrals to community-based agencies for ongoing support.  

Treatment for HIV:  Currently at the UMLAZI Section D ANC all HIV-positive pregnant women are offered the HIVNET 012 single dose Nevirapine protocol for prevention of mother-to-child transmission.  The protocol  includes treatment of opportunistic infections and prophylaxis.  The clinical care and drugs are at no cost to the mothers and infants and includes partners where available.  All HIV positive women and their infants are provided ongoing care at the Clinic.  Antiretroviral therapy (HAART) is available to HIV infected women with CD4 < 200 or Clinical Stage IV and their infants if also HIV infected. Women and children who are eligible for HAART are screened and receive adherence counseling at the SED Clinic. HAART is initiated at PMMH and women receive continuum of care at the clinic. Currently, about 5% of the patients at the Clinic are on potent anti-retroviral therapy that is accessed through their private health insurance or through participation in industry-sponsored trials currently underway at the Clinic and about 50 women have been prescribed ARVs through PMMH following delivery. 

Description of risks and strategies to minimize risk: 
Women who participate in this study may be exposed to some psychosocial risks related to: 1) Negative outcomes of disclosure:  It is possible that women who share HIV test results with their sexual partners will experience negative reactions.  This risk is one that all women in the ANC clinic face, regardless of enrollment in this study.  We will carefully train the HIV counselors to assess whether it is safe for women to share HIV test results with her sexual partners.  The enhanced counseling is designed to help women carefully weigh the costs and benefits to disclosure.  Counselors use a structured discussion counseling tool with to help women assess the risk of violence following disclosure.  If women report a history of violence, anticipate negative reactions from their partner when they disclose and still intend to disclose, counselors will actively encourage women to consider alternative options for disclosing, including postponing disclosure until a time when their safety can be insured, or considering a mediated form of disclosure in which they bring their partner to the clinic to disclose in the presence of a counselor, or they identify a trusted family member or friend who can be with them when they disclose to their partner at home.  2) Precipitation of emotional crisis:  It is possible that the qualitative and survey interviews will precipitate an emotional crisis among women who have not had the opportunities to talk about these emotionally charged topics in the past.  To address this risk the interviewers will be trained to respond empathetically to the women.  However, since interviewers are not trained counselors, they will also be trained to refer women who need additional support to the HIV counselors in the clinic.  If the HIV counselors identify women who express psychological needs beyond those that they are trained to handle, they will refer these clients to the Psychologists and Psychiatrists at PMMH.  The Psychologists and Psychiatrists that staff PMMH provide ongoing psychological support for all clients.  These services will be available to all women in this trial.  An active referral mechanism to these support groups will be part of the intervention protocol tested in this trial.    


Description of Benefits:  There are no direct benefits to women who participate in this study.  Women may benefit from knowing that the information learned from this study will help to improve counseling services for other women like themselves.  Women may also benefit from spending more time with the counselor talking about and developing a plan for disclosure.  The trained counselor will help women to assess support systems that may be of assistance to women through the disclosure process.  Women in both arms will also be invited to return to the counselors as often as needed throughout the study period.  In terms of social benefits, if the trial is successful there are potentially enormous social benefits that can be gained from the study.  We expect this intervention to be effective, cost-effective, sustainable and easily disseminated in other developing country settings.  Prevention of mother to child transmission of HIV is one of the few areas in the field of HIV prevention that has had enormous success.  There is a global call to extend the benefits of HIV treatment for PMTCT to developing countries that bear the largest burden of the epidemic.  The intervention we are proposing to test through this proposal is designed to maximize the efficiency and efficacy of PMTCT programs.  
  
IRB approvals: Approval for this study will be sought from the University of North Carolina, Johns Hopkins Committee on Human Research and the Institutional Review Boards at the Nelson R. Mandela School of Medicine.  The IRB at the Nelson R. Mandela School of Medicine already has a U.S. Federal Wide Assurance (number: 00000678). 

Inclusion of Women: Participants in this trial are all women.  The proposed study is designed to address the psychosocial needs of women who are tested for HIV as part of their antenatal care services.  

Inclusion of Minorities: The trial is being conducted in the antenatal clinic of Section D  in Umlazi Durban, South Africa.  This is a public antenatal health clinic that serves primarily women of low socioeconomic status from the surrounding communities.  The sample for this study will reflect the demographics of the clients seeking services at the UMLAZI SED ANC clinic.  We anticipate that the majority of participants will be Black-African (95%), with a smaller proportion Asian (3%) and White (2%) as well.  The trial design calls for a random sample of women from the ANC clinic. Thus, any tribal groups seeking ANC services at Section D clinic  will be eligible for participation in this trial.  

Inclusion of Children: This trial involves participants between the ages of 18 and 21.  Under South Africa law individuals 18 years or older can independently consent to participate in a research study without the consent of a parent.  


F. 	LITERATURE CITED 
1. The President's Emergency Plan for AIDS Relief. http://www.whitehouse.gov/news/releases/2003/07/20030702-4.html ed, 2004.
2. South African Department of Health. Demographic and Health Survey: Chapter 7, 1998.
3. Moodley D. Progress Report of PMTCT Programme in Kwazulu Natal. 2002.
4. Health Systems Trust Reports. South African Health Review, 2001.
5. South African Departments of Health and Treasury. South African Joint Health and Treasury Task Team Report, 2003.
6. South African Ministry of Health. National HIV and Syphilis Antenatal Sero-Prevalence Survey in South Africa, 2002.
7. Human Sciences Research Council. Nelson Mandela/HSRC Study of HIV/AIDS Household Survey 2002, 2002.
8. Sturm A, Wilkinson, D.,  Ndovela, N., Bowen, S., Connolly, C. Pregnant women as a reservoir of undetected sexually transmitted diseases in rural South Africa: Implications for disease control. American Journal of Public Health 1998;88(8):1243-1245.
9. Dabis F, Msellati P, Meda N, et al. 6-month efficacy, tolerance, and acceptability of a short regimen of oral zidovudine to reduce vertical transmission of HIV in breastfed children in Cote d'Ivoire and Burkina Faso: a double-blind placebo-controlled multicentre trial. DITRAME Study Group. DIminution de la Transmission Mere-Enfant. Lancet 1999;353(9155):786-92.
10. Wiktor SZ, Ekpini E, Karon JM, et al. Short-course oral zidovudine for prevention of mother-to-child transmission of HIV-1 in Abidjan, Cote d'Ivoire: a randomised trial. Lancet 1999;353(9155):781-5.
11. Shaffer N, Chuachoowong R, Mock PA, et al. Short-course zidovudine for perinatal HIV-1 transmission in Bangkok, Thailand: a randomised controlled trial. Bangkok Collaborative Perinatal HIV Transmission Study Group. Lancet 1999;353(9155):773-80.
12. Guay LA, Musoke P, Fleming T, et al. Intrapartum and neonatal single-dose nevirapine compared with zidovudine for prevention of mother-to-child transmission of HIV-1 in Kampala, Uganda: HIVNET 012 randomised trial. Lancet 1999;354(9181):795-802.
13. Tsague LNN, A.; Engozo;o, A.; Monny, M.; Ayouba, A.; Nerrienet, E.; Tene, G.; Eteki, N.; Nkam, M.; et al. Three years of the National PMTCT program in Cameroon:  From a Pilot to a National Public Health program. XV International AIDS Meeting 2004, Bangkok, Thailand.
14. Washington SB, G. Barriers to uptake of prevention of mother-to-child transmission of HIV services in Lusaka, Zambia: A qualitative study. XV International AIDS Meeting 2004, Bangkok, Thailand. 
15. Mayer AG, H.; Mugenyi, K.' Mbezi, P.; Odera, J.; Masanja, B.; Simo, S.; Ali, M.; Weidenhammer, A.; Karcher, H.; Kabasinguzi, R. Uptake of PMTCT/MTCT plus programs in Kenya, Tanzania and Uganda. XV International AIDS Meeting 2004, Bangkok, Thailand.
16. Nagadya AN, R.; Mbulaiteye, S.M.; Wabwire-Mangen, F.; Kakitahi, T.J. Challenges and Opportunities to implementation of mother to child HIV transmission preventive services in rural Uganda. XV International AIDS Meeting 2004, Bangkok, Thailand.
17. Wakabi Waisai T. Barriers to the provision of PMTCT services in Masaka district, Uganda. XV International AIDS Meeting 2004, Bangkok, Thailand.
18. Guinan ME, Leviton L. Prevention of HIV infection in women: overcoming barriers. J Am Med Womens Assoc 1995;50(3-4):74-7.
19. Manhart LE, Dialmy A, Ryan CA, Mahjour J. Sexually transmitted diseases in Morocco: gender influences on prevention and health care seeking behavior. Soc Sci Med 2000;50(10):1369-83.
20. Molyneux CS, Murira G, Masha J, Snow RW. Intra-household relations and treatment decision-making for childhood illness: a Kenyan case study. J Biosoc Sci 2002;34(1):109-31.
21. Maman S, Mbwambo J, Hogan NM, Kilonzo GP, Sweat M. Women's barriers to HIV-1 testing and disclosure: challenges for HIV-1 voluntary counselling and testing. AIDS Care 2001;13(5):595-603.
22. Population Council. Evaluation of United Nations supported pilot projects for the prevention of mother-to-child transmission of HIV: UNICEF, 2003: 54.
23. Burke MGRR, M. Maximizing male participation in prevention of mother to child transmission programs in Tanzania. XVII International AIDS Conference 2004, Bangkok, Thailand.
24. Tonwegold BE, D.; Viho, I; Toure, S.; Kone, M.; Ehouo, B.; Bequet, L.; Sihe, A.; Leroy, V.; Dabis, F.; Abrams, E.J. Where are the men? Involvement of male partners in a family centred care program in Abidjan,Côte d'Ivoire. XVII International AIDS Conference 2004, Bangkok, Thailand.
25. Mbuyi BM, R.; Vaz, L.M.; Callens, S.; Behets, F.; Kokolomami, J. Involving male partners in programs to prevent mother-to-child transmission of HIV in Kinshasa, DRC. XVII International AIDS Conference 2004, Bangkok, Thailand.
26. Bunjo NL. View of HIV-infected mothers on early cessation of breastfeeding in Kampala. XIV International AIDS Conference 2002, Barcelona.
27. Bland RM RV, Coovadia HM. Infant feeding choices of HIV-infected mothers compared with their home circumstances. XIV International AIDS Conference 2002, Barcelona.
28. Desclaux A TB, ed. Allaitement et vih en Afrique de L'ouest: de l'anthropologie a la sante publique, 2000.
29. Dauossi RD. Feeding infants when mothers are HIV positive in African settings: can communities be mobilized to support a mother's decision? XIV International AIDS Conference 2002, Barcelona.
30. Antelman G, Smith FMC, Kaaya S, et al. Predictors of HIV-1 serostatus disclosure: a prospective study among HIV-infected pregnant women in Dar es Salaam, Tanzania. Aids 2001;15(14):1865-74.
31. Kilewo C, Massawe A, Lyamuya E, et al. HIV counseling and testing of pregnant women in sub-Saharan Africa: experiences from a study on prevention of mother-to-child HIV-1 transmission in Dar es Salaam, Tanzania. J Acquir Immune Defic Syndr 2001;28(5):458-62.
32. Nebie Y, Meda N, Leroy V, et al. Sexual and reproductive life of women informed of their HIV seropositivity: a prospective cohort study in Burkina Faso. J Acquir Immune Defic Syndr 2001;28(4):367-72.
33. Farquhar C, Mbori ND, Bosire R, Nduati R, Kreiss J, John G. Prevalence and correlates of partner notification regarding HIV-1 in an antenatal setting in Nairobi, Kenya. Int Conf AIDS 2000;13.
34. Gaillard P, Meilis R, Mwanyumba F, et al. Consequences of announcing HIV seropositivity to women in an African setting: lessons for the implementation of HIV testing and interventions to reduce mother-to-child HIV transmission. Int Conf AIDS 2000;13.
35. van der Straten A., King R, Grinstead O, Serufilira A, Allen S. Couple communication, sexual coercion and HIV risk reduction in Kigali, Rwanda. Aids 1995;9(8):935-44.
36. Maman S MJ, Hogan M, Kilonzo GP, Weiss E, Sweat M. High rates and positive outcomes of HIV serostatus disclosure to sexual partners: reasons for cautious optimism from a VCT clinic in Dar Es Salaam, Tanzania. AIDS and Behavior;7(4):373-382.
37. Keogh P, Allen S, Almedal C, Temahagili B. The social impact of HIV infection on women in Kigali, Rwanda: a prospective study. Soc Sci Med 1994;38(8):1047-53.
38. Grinstead OA, Gregorich SE, Choi KH, Coates T. Positive and negative life events after counselling and testing: the Voluntary HIV-1 Counselling and Testing Efficacy Study. Aids 2001;15(8):1045-52.
39. Lie GT, Biswalo PM. HIV-positive patient's choice of a significant other to be informed about the HIV-test result: findings from an HIV/AIDS counselling programme in the regional hospitals of Arusha and Kilimanjaro, Tanzania. AIDS Care 1996;8(3):285-96.
40. MacNeil JM, Mberesero F, Kilonzo G. Is care and support associated with preventive behaviour among people with HIV? AIDS Care 1999;11(5):537-46.
41. Simoni J, Pantolone, DW. Secrets and Safety in the Age of AIDS:  Does HIV Disclousre lead to Safer Sex? Topics in HIV Medicine 2004;12(4):109-118.
42. Basani LO, F.M.; Namande, J.; Nayiga, I.S.; Esiru, G.; Kituuka, P.; Okong, P. Experiences screening clients for MTCT Plus programme in Kampala, Uganda. XV International AIDS Meeting 2004, Bangkok, Thailand.
43. Issiaka S, Cartoux M, Ky-Zerbo O, et al. Living with HIV: women's experience in Burkina Faso, West Africa. AIDS Care 2001;13(1):123-8.
44. Heyward WL, Batter VL, Malulu M, et al. Impact of HIV counseling and testing among child-bearing women in Kinshasa, Zaire. Aids 1993;7(12):1633-7.
45. Koenig LJ, Moore J. Women, violence, and HIV: a critical evaluation with implications for HIV services. Matern Child Health J 2000;4(2):103-9.
46. Mathews C, Kuhn L, Fransman D, Hussey G, Dikweni L. Disclosure of HIV status and its consequences. S Afr Med J 1999;89(12):1238.
47. Jewkes R, Penn-Kekana L, Levin J, Ratsaka M, Schrieber M. Prevalence of emotional, physical and sexual abuse of women in three South African provinces. S Afr Med J 2001;91(5):421-8.
48. World Health Organization. Treating 3 million people by 2005: Making it Happen, The WHO Strategy, 2003.
49. Sweat MD ORK, Schmid GP, Denison J, De Zoysa I. Cost-effectiveness of nevirapine to prevent mother-to-child HIV transmission in eight African countries. AIDS 2004;18(12):1661-71.
50. Stover J. The benefits of providing family planning counseling within PMTCT services. Consultative meeting on the linkages between reproductive health and HIV/AIDS: family planning and prevention of mother to child transmission. 2004, Glion, Montreaux.
51. Delvaux TL, M. Opportunities for linkages with sexual and reproductive health from the perspective of HIV/AIDS programs. Consultative meeting on the linkages between reproductive health and HIV/AIDS: family planning and prevention of mother to child transmission. Glion, Montreaux, 2004.
52. Cates WJ. Contraceptive choices for HIV-infected women. Consultative meeting on the linkages between reproductive health and HIV/AIDS: family planning and prevention of mother to child transmission 2004, Glion, Montreaux.
53. Reynolds HWJ, B.; Homan, R.; Johnson, L. Cost-effectiveness analysis of two interventions to avert HIV-positive births. Consultative meeting on the linkages between reproductive health and HIV/AIDS: family planning and prevention of mother to child transmission 2004, Glion, Montreaux.
54. Rutenberg N. Review of program experiences with integrating family planning and prevention of mother to child transmission. Consultative meeting on the linkages between reproductive health and HIV/AIDS: family planning and prevention of mother to child transmission 2004, Glion, Montreaux.
55. Efficacy of voluntary HIV-1 counselling and testing in individuals and couples in Kenya, Tanzania, and Trinidad: a randomised trial. The Voluntary HIV-1 Counseling and Testing Efficacy Study Group. Lancet 2000;356(9224):103-12.
56. World Health Organization. Technical consultation on voluntary HIV counseling and testing: models for implementation and strategies for scaling up of VCT services, 2001.
57. Health Systems Trust. Interim Findings on the National PMTCT Pilot Sites: Lessons and Recommendations, 2002.
58. James KW, S.; Struthers, H.; McIntyre, J.A. Development of a toolkit for use in HIV/AIDS support group facilitation and education. XV International AIDS Meeting 2004, Bangkok, Thailand.
59. Sweat M, Gregorich S, Sangiwa G, et al. Cost-effectiveness of voluntary HIV-1 counselling and testing in reducing sexual transmission of HIV-1 in Kenya and Tanzania. Lancet 2000;356(9224):113-21.
60. Maman S, Mbwambo JK, Hogan NM, et al. HIV-positive women report more lifetime partner violence: findings from a voluntary counseling and testing clinic in Dar es Salaam, Tanzania. Am J Public Health 2002;92(8):1331-7.
61. Petra TST. Efficiency of three short-course regimens of zidovudine and lamivudine in preventing early and late transmission on HIV-1  from mother to child in Tanzania, South Africa, and Uganda (Petra study): a randomized, double-blind, placebo-controlled trial. Lancet 2002;359:1176-1186.
62. Moodley D MJ, Coovadia H, Gray G, McIntyre J, Hofmyer J, Nikodem C, Hall D, Gigliotti M, Robinson P, Boshoff L, Sullivan JL. A multicenter randomized controlled trial of nevirapine versus a combination of zidovudine and lamivudine to reduce intrapartum and early postpartum mother-to-child transmission of human immunodeficiency virus type 1. Journal of Infectious Diseases 2003;187(5):725-35.
63. Gaillard P. FMG, Dabis F., Coovadia H., et al. Use of antiretroviral drugs to prevent HIV-1 transmission through breast feeding: from animal studies to randomized clinical trials. J Acquir Immune Defic Syndr 2004;35:178-187.
64. Coovadia H. Infant feeding and prevention of mother-to-child HIV transmission. SAMJ 2004;94(7):496-497.
65. Coutsoudis A, Pillay K, Spooner E, Kuhn L, Coovadia HM. Influence of infant-feeding patterns on early mother-to-child transmission of HIV-1 in Durban, South Africa: a prospective cohort study. South African Vitamin A Study Group. Lancet 1999;354(9177):471-6.
66. Department of Health, Govenment of South Africa. Feeding of infants of HIV-positive mothers: HIV/AIDS Policy Guideline, 2000.
67. Moodley D. PMTCT Site Manual. 2nd Edition ed.
68. Des Jarlais DP, D.; Milliken, J. Audio-computer interviewing to measure risk behaviour for HIV among injecting drug users:  a quasi-randomised trial. The Lancet 1999;9165:1657-1671.
69. Turner CK, L, Rogers, S.M.; Linderg, L.D.; Pleck, J.H.; Sonenstein, F.L. Adolescent behavior, drug use and violence: increased reporting with computer survey technology. Science 1998;280:867-873.
70. Hewett PC, Mensch, B.S., Erulkar, A.S. Consistency in reporting of sexual behavior by adolescent girls in Kenya: a comparison of interviewing methods. Sexually Transmitted Infections 2004;80:43-48.
71. van de Wijgert J, Padian, N, Shiboski, S, Turner, C. Is audio computer-assisted self-interviewing a feasible method of surveying in Zimbabwe? Int J Epidemiol 2000;29(5):885-890.
72. Fishbein M, Pequegnat W. Evaluating AIDS prevention interventions using behavioral and biological outcome measures. Sex Transm Dis 2000;27(2):101-10.
73. Moodley P, Martin IM, Ison CA, Sturm AW. Typing of Neisseria gonorrhoeae reveals rapid reinfection in rural South Africa. J Clin Microbiol 2002;40(12):4567-70.
74. Krieger J, Alderete J. Trichomonas vaginalis and Trichomoniases. In: Holmes K, Sparling P, Mardh P-A, et al., eds. Sexually Transmitted Diseases. New York: McGraw Hill, 1999.
75. Koblavi-Deme SM, C.; Yaroow, D. et al. Sensitivity and specificity of human immunodeficiency virus rapid serologic screening assays and testing algorithms in antenatal clinics in Abijan, Ivory Coast. Journal of Clinical Microbiology 2001;39(5):1808-1812.
76. Ketema TZ, C.; Edelman, D.C.; Saville, R.; Constatine, N.T. Assessment of the performance of rapid testing lateral flow assay for detection of antibodies to HIV. Journal of Acquired Immune Deficiency Syndrome 2001;27(1):63-70.
77. Andersson SdZN, H.; Biberfeld, G. Field evaluation of alternative testing strategies for diagnosis and differentiation of HIV-1 and HIV-2 infection in an HIV-1 and HIV-2 prevalent area. AIDS 1997;11(15):1815-22.
78. Kassler WJH, C.; Jones, W.K.; Kennedy, E.J.; George, J.R. Performance of rapid onsite human immunodeficiency virus antibody assay in public health settings. Journal of Clinical Microbiology 1995;33(11):2899-902.
79. French NN, B.; Namara, A.H.; Kyalo, G. HIV testing strategies at a community clinic in Uganda. AIDS 1997;11(14):1779-80.
80. Ruff A. personal communication, April 17, 2005.
81. Langford CP, Bowsher J, Maloney JP, Lillis PP. Social support: a conceptual analysis. J Adv Nurs 1997;25(1):95-100.
82. Shumaker SA BA. Toward a theory of social support: closing conceptual gaps. Journal of Social Issues 1984;40(4):11-36.
83. Cohen S UL, Gottlieb BH. Social support measurement and intervention: a guide for health and social scientists: Oxford University Press.
84. House JS UD, Landis KR. Structures and processes of social support. Annual Review of Sociology 1988;14:293-318.
85. Glanz K LF, Rimer B. Health behavior and health education: theory, research, and practice: Jossey-Bass Publishers, 1996.
86. Heise LE, C. Transforming AIDS prevention to meet women's needs: a focus on developing countries. Social Science and Medicine 1995;40(7):931-43.
87. Abdool Karim Q. Barriers to preventing Human Immunodeficiency Virus in Women: Experiences from KwaZulu-Natal, South Africa. Journal of the American Women's Association 2001;56:193-6.
88. Sweat MOD, C.; O'Donnell, L. Cost-effectiveness of a brief video-based HIV intervention for African-American and Latino sexually transmitted disease clinic clients. AIDS 2001;15:781-787.
89. CDC. Revised guidelines for HIV counseling, testing and referral. MMWR Morb Mortal Wkly Rep 2002;50(RR-19):1-57.
90. UNAIDS. Counselling and HIV/AIDS: UNAIDS Best Practices Collection. Geneva: UNAIDS, 1997.
91. Kalichman S RD, DiFonzo K, Simpson D, Kyomugisha F, Austin J, Luke W. Initial development of scales to assess self-efficacy for disclosing HIV status and negotiating safer sex in HIV-positive persons. AIDS and Behavior 2001;5(3):291-296.
92. UNAIDS. Counseling and Voluntary Testing for HIV Infection in Antenatal Care:  Practical Considerations for Implementation, 1999.
93. World Health Organization. Voluntary Counseling and testing for HIV Infection in Antenatal Care:  Practical Considerations for Implementation, 1999.
94. WHO Techical Consultation on Behalf of the UNFPA/UNICEF/WHO/UNAIDS Inter-Agency Task Team on Mother-to-Child Transmission of HIV. New Data on the Prevention of Mother-to-child Transmission of HIV and their Policy Implications:  Conclusions and Recommendations, 2001.
95. World Health Organization. Increasing access to HIV testing and counseling: Report of a WHO consultation. Geneva, Switzerland: WHO, 2002.
96. Landis JR, Koch, GG. The measurement of observer agreement for categorical data. Biometrics 1977;33:159.
97. Metzger DSK, B.; Turner, C.; Navaline, H.; Valenti, F.; Holte, S.; Gross, et al. Randomized-controlled trial of audio-computer assisted self-interviewing: utility and acceptability in longtudinal studies. america Journal of Epidemiology 2000;152(2):99-106.
98. Macalino GEC, D.D.; Latkin, C; Strathdee, S.A.; Vlahov, D. Risk behaviors by audio-computer assisted self-interview among HIV-seropositive and HIV-seronegative injection drug users. AIDS Educ Prev 2002;14(5):367-378.
99. Mensch BSH, P.C.; Erulkar, A.S. The reporting of sensitive behavior by adolescents:  a methdological experiment in Kenya. Demography 2003;40(2):247-268.
100. Allen DRC, J.W.; Manopaiboon, C.; Jenkins, R.A.; Uthaivoravit, W.; Kilmarx, P.H.; van Griensven, F. Sexual health risks among young Thai women:  Implications for HIV/STD prevention and contraception. AIDS and Behavior 2003;7(1):9-21.
101. Pequegnat W, Fishbein M, Celentano D, et al. NIMH/APPC workgroup on behavioral and biological outcomes in HIV/STD prevention studies: a position statement. Sex Transm Dis 2000;27(3):127-32.
102. De Vicenzi I. A longitudinal study of human immunodeficiency virus transmission by heterosexual partners. New England Journal of Medicine 1994;331:341-346.
103. National Institute of Allergy and Infectious Diseases.  Workshop Summary: Scientific Evidence on Condom Effectiveness  for Sexually Transmitted Disease (STD) Prevention. Bethesda: National Institutes of Allergy and Infectious Diseases.
104. Saracco AM, M.; Nicolosi, A. et al. Man-to-woman sexual transmission of HIV: longitudinal study of 343 steady partners of infected men. J Acquir Immune Defic Syndr 1993;6:497-502.
105. Ross JAW, W.L. International Family Planning Perspectives 2001;27(1):20-27.
106. Serovich JM, Kimberly JA, Mosack KE, Lewis TL. The role of family and friend social support in reducing emotional distress among HIV-positive women. AIDS Care 2001;13(3):335-41.
107. Wilcox BL. Social support, life stress, and psychological adjustment: a test of the buffering hypothesis. Am J Community Psychol 1981;9(4):371-86.
108. Wilcox BL. Social support in adjusting to marital disruption. In: Gottlieb B, ed. Social Networks and Social Support. Beverley Hills, CA: Sage Publications, 1981: 97-115.
109. Kaaya S S-FM, Mbwambo J, Lee B, Msamanga G, Fawzi W. Validty and calibration of the Kiswahili version of the Hopkins symptom checklist--25 amongst HIV-positive antentatal clinic attendees in Dar Es Salaam, Tanzania. Acta Psychiatrica Scandinavica;106(1):9-19.
110. Jewkes R LJ, Penn-Kekana L. Risk factors for domestic violence: findings from a South African cross-sectional survey. In Press: Social Science and Medicine 2002.
111. Straus M HS, Boney-McCoy S, Sugarman D. The revised conflict tactic scales (CTS2): development and preliminary psychometric data. Journal of Family Issues 1996;17(3):283-316.
112. Garcia-Moreno C. Preliminary results of multi-country study on domestic violence and women's health. Presentation at PATH 2002, Washington, D.C.
113. World Health Organization. Putting women first:  Ethical and safety recommendations for research on violence against women. Geneva: World Health Organization, 2001.
114. Patton MQ. Desiging Qualitative Studies. Qualitative Evaluation and Research Studies. Newbury Park, CA: Sage Publications, 1990: 169-186.
115. Antelman GS, FMC; Kaaya, S. et al. Predictors of HIV-1 serostatus disclosure: a prospective study among HIV-infected pregnant women in Dar es Salaam, Tanzania. AIDS 2001;154(14):1865-74.
116. Population Council Horizons Program Report. Prevention of mother-to-child transmission of HIV: Population Council, 2002.
117. Pettifor AE. Sexual Power and HIV Risk, South Africa. Emerg Infect Dis 2004;10(11):1996-2004.
118. Kamb ML, Fishbein M, Douglas JM, Jr., et al. Efficacy of risk-reduction counseling to prevent human immunodeficiency virus and sexually transmitted diseases: a randomized controlled trial. Project RESPECT Study Group. Jama 1998;280(13):1161-7.
119. McCoy D, Besser M, Visser R, Doherty T. Interim findings on the national PMTCT pilot sites: lessons and recommendations. Health Systems Trust Report, 2002.
120. Dunkle KL, Jewkes RK, Brown HC, Gray GE, McIntryre JA, Harlow SD. Gender-based violence, relationship power, and risk of HIV infection in women attending antenatal clinics in South Africa. Lancet 2004;363(9419):1415-21.
121. Bernard H. Research Methods in Anthropology:  Qualitative and Quantitative Approaches. Thousand Oaks, California: Sage Publications, 2000.
122. Miles M, Huberman, MA. Qualitative data analysis: An expanded sourcebook. London: Sage Publications, 1994.


 
